# Nitrogen-embedded buckybowl and its assembly with C_60_

**DOI:** 10.1038/ncomms9215

**Published:** 2015-09-04

**Authors:** Hiroki Yokoi, Yuya Hiraoka, Satoru Hiroto, Daisuke Sakamaki, Shu Seki, Hiroshi Shinokubo

**Affiliations:** 1 Department of Applied Chemistry, Graduate School of Engineering, Nagoya University, Nagoya 464-8603, Japan; 2 Department of Molecular Engineering, Graduate School of Engineering, Kyoto University, Kyoto 615-8510, Japan

## Abstract

Curved *π*-conjugated molecules have attracted considerable interest because of the unique properties originating from their curved *π* surface. However, the synthesis of such distorted molecules requires harsh conditions, which hamper easy access to heteroatom-containing curved *π* systems. Here we report the synthesis of a π-extended azacorannulene with nitrogen in its centre. The oxidation of 9-aminophenanthrene provides tetrabenzocarbazole, which is converted to the azabuckybowl through palladium-catalysed intramolecular coupling. The electron-donating nature and curved *π* surface of the azabuckybowl enable its tight association with C_60_ in solution and solid states. High charge mobility is observed for the azabuckybowl/C_60_ assembly. This compound may be of interest in the fields of curved *π* systems as fullerene hosts, anisotropic π donors and precursors to nitrogen-containing nanocarbon materials.

## Abstract (web summary)

Curved *π*-conjugated molecules have potentially interesting chemical and electronic properties but the harsh conditions required for their synthesis can make it difficult to incorporate heteroatoms. Here, the authors prepare a *π*-extended azacorannulene with nitrogen in its centre.

## Graphical abstract

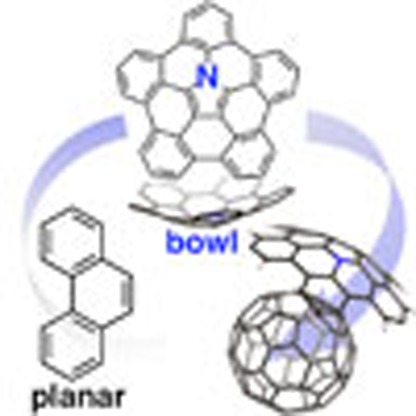

Curved *π*-conjugated molecules have captivated numerous scientists^1^,^2^,^3^,^4^. Curving a *π* system induces a large displacement from a plane to construct three-dimensional structures^5^,^6^,^7^,^8^,^9^,^10^. Besides their figurative beauty, the curved *π* surface generates unique functions such as chiroptical properties, anisotropic electron transitions and dynamic motion in solution and solid states^11^,^12^,^13^. To enhance these characteristics, the introduction of heteroatoms is an effective strategy. However, the synthesis of heteroatom-containing curved *π* systems remains a challenge^14^,^15^. The preparation of distorted *π* systems requires harsh reaction conditions that do not tolerate heterocyclic molecules.

Buckybowls, that is, bowl-shaped molecules such as corannulenes^16^ and sumanenes^17^ represent important curved *π*-conjugated molecules, which can be precursors for the bottom-up synthesis of fullerenes and nanotubes. In 1966, Barth and Lawton^16^ reported the first chemical synthesis of corannulene. Since Scott and Siegel's groups^18^,^19^,^20^,^21^ developed their straightforward synthesis of corannulene, numerous bowl-shaped hydrocarbons have been synthesized. On the other hand, the nitrogen-embedded bowl-shaped molecules have been sought as model compounds for azafullerenes and nitrogen-doped carbon nanotubes^22^,^23^,^24^. Furthermore, dramatic changes in electronic structures of buckybowls by nitrogen are expected. However, the synthesis of buckybowls with internal nitrogen atoms has been still challenging^25^. Oxidative fusion approaches are not compatible with electron-rich nitrogen because of its less tolerant nature for oxidation.

Recently, our group reported the oxidative dimerization of aminoarenes to distorted *π*-conjugated molecules in a one-step operation^26^,^27^. We here disclose that the oxidation of 9-aminophenanthrenes affords tetrabenzocarbazoles in good yields. Furthermore, consecutive fusion reactions of tetrabenzocarbazole **2** through palladium-catalysed C–H/C–Cl and C–H/C–Br coupling achieve the synthesis of nitrogen-embedded buckybowl **5**, that is, ‘azabuckybowl'^28^ under mild conditions. Owing to the electron-donating nature of the nitrogen atom, azabuckybowl **5** strongly interacts with C_60_ to furnish an inclusion complex, which exhibits a substantially high charge-carrier mobility in the solid state.

## Results

### Synthesis of nitrogen-embedded buckybowl

**Figure 1:**
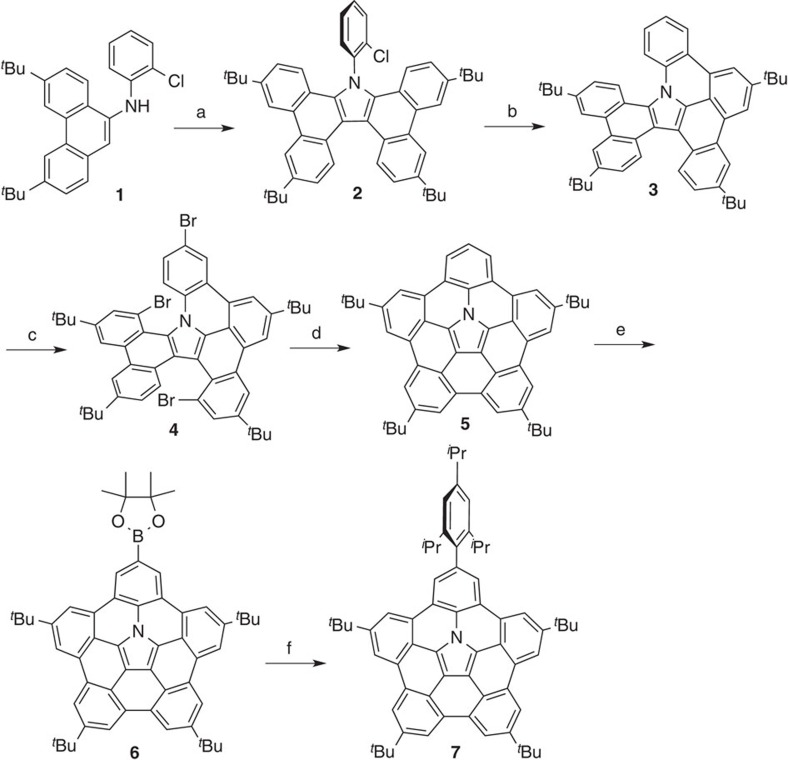
Synthesis of nitrogen-embedded buckybowl 5 from phenanthrene 1. Conditions: (a) 2,3-dichloro-5,6-dicyanobenzoquinone (DDQ), TFA, toluene, room temperature, 1 h, 94% yield. (b) Pd(OAc)_2_, PCy_3_·HBF_4_, K_2_CO_3_, DMA, 130 °C, 43 h, 63% yield. (c) Br_2_, CCl_4_, 70 °C, 12.5 h, 56% yield. (d) Pd(OAc)_2_, PCy_3_·HBF_4_, K_2_CO_3_, DMA, 130 °C, 16 h, 46% yield. (e) bis(pinacolato)diboron, [Ir(OMe)cod]_2_, 4,4'-di-*tert*-butyl-2,2'-bipyridyl, octane, 10.5 h, 110 °C, 80% yield. (f) 2-bromo-1,3,5-triisopropylbenzene, PdCl_2_(dppf)·CH_2_Cl_2_, Cs_2_CO_3_, 1,4-dioxane, 13 h, 100 °C, 55% yield.

The synthesis of nitrogen-embedded buckybowl **5** started with the oxidative dimerization of **1** (Fig. 1)^26^. 9-Aminophenanthrene **1** was oxidized to tetrabenzocarbazole **2** in 94% yield. Reaction of **2** with Pd(OAc)_2_/tricyclohexylphosphine provided singly fused product **3** in 63% yield^29^. The twisted conformation of **3** was unambiguously elucidated by X-ray diffraction analysis (Supplementary Fig. 18). The bromination of **3** with bromine afforded tribrominated product **4** in 56% yield. Finally, the palladium-catalysed double C–H/C–Br coupling furnished nitrogen-embedded buckybowl **5** in 46% yield. The proton nuclear magnetic resonance (^1^H NMR) spectrum of **5** exhibited six proton signals in the aromatic region, indicating the formation of a fused and symmetrical molecule.

### Structural elucidation and characteristics of azabuckybowl

**Figure 2:**
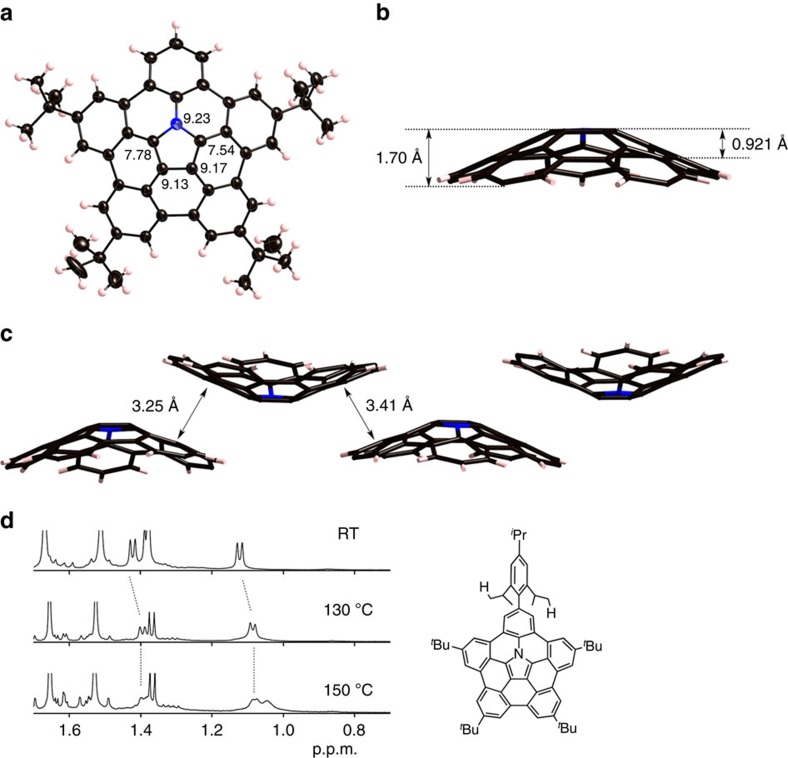
Structural features of azabuckybowls. (**a**) Top view and POAV pyramidalization angles, (**b**) side view of **5** and (**c**) packing structure of **5** in the crystal. Thermal ellipsoids in **a** are scaled at 50% probability level and *t*-butyl groups are omitted for clarity in **b** and **c**. (**d**) Temperature-dependent NMR spectra of **7** in 1,2-dichlorobenzene-*d*_4_. POAV angles and bowl depths in one of two molecules in the crystal are displayed in **a** and **b**. Solvent molecules (*o*-xylene) in the crystal structure of **5** were omitted for clarity. RT, room temperature.

The bowl-shaped structure of **5** was unambiguously elucidated by X-ray diffraction (Fig. 2). In the crystal, one asymmetric unit contained two independent molecules of **5**. The bowl depth, which is defined as the distance between the mean plane that consisted of five carbons at the edge and the centroid of the pyrrole ring, was 1.65 and 1.70 Å. The bowl depth of the central azacorannulene core was 0.90 and 0.92 Å, which is slightly greater than that of corannulene (0.86 Å). The curvature of **5** was further evaluated by Haddon's *π*-orbital axis vector (POAV) angles^30^. As shown in Fig. 2a, the POAV angles around the central pyrrole ring are in the range of 7.2°–9.3°. These values are comparable with that of corannulene (9.1°). It is noteworthy that the molecules constructed a one-dimensional chain stacking structure in the crystal (Fig. 2c). Distances between the two closest molecules were 3.25 and 3.41 Å, indicating the existence of a *π*–*π* interaction.

Bowl-to-bowl inversion of the azabuckybowl was investigated. Azabuckybowl **5** was further functionalized by iridium-catalysed C–H borylation to provide **6** in 80% yield^31^,^32^. The Suzuki–Miyaura cross-coupling reaction of **6** with 2-bromo-1,3,5-triisopropylbenzene furnished the corresponding coupling product **7** in 55% yield. The ^1^H NMR spectrum of **7** in 1,2-dichlorobenzene-*d*_4_ at room temperature exhibited three doublet peaks for methyl protons of isopropyl groups at 1.43, 1.39 and 1.13 p.p.m. This non-symmetric feature indicates that **7** shows no bowl-to-bowl inversion at room temperature. As the temperature was raised, two proton signals at 1.43 and 1.13 p.p.m. were gradually broadened (Fig. 2d). Even at 170 °C, these signals were not coalesced. Accordingly, the bowl-to-bowl inversion energy (*ΔG*^‡^) was measured by two-dimensional exchange spectroscopy (2D EXSY) experiments. At 393 K, *ΔG*^‡^ was determined to be 23.3 kcal mol^−1^ in 1,2-dichlorobenzene-*d*_4_ (Supplementary Figs 32 and 33). This value is higher than that of the parent sumanene (*ΔG*^‡^=19.7–20.4 kcal mol^−1^)^33^. The high bowl-inversion energy of **7** was also supported by theoretical calculations. The inversion barrier of **7** was calculated to be 19.9 kcal mol^−1^ by density functional theory (DFT) calculations at the B3LYP/cc-pVDZ level, which is higher than those of sumanene (18.2 kcal mol^−1^) and corannulene (9.1 kcal mol^−1^) calculated at the same level of theory^34^.

### Optical and electrochemical properties of azabuckybowl

**Figure 3:**
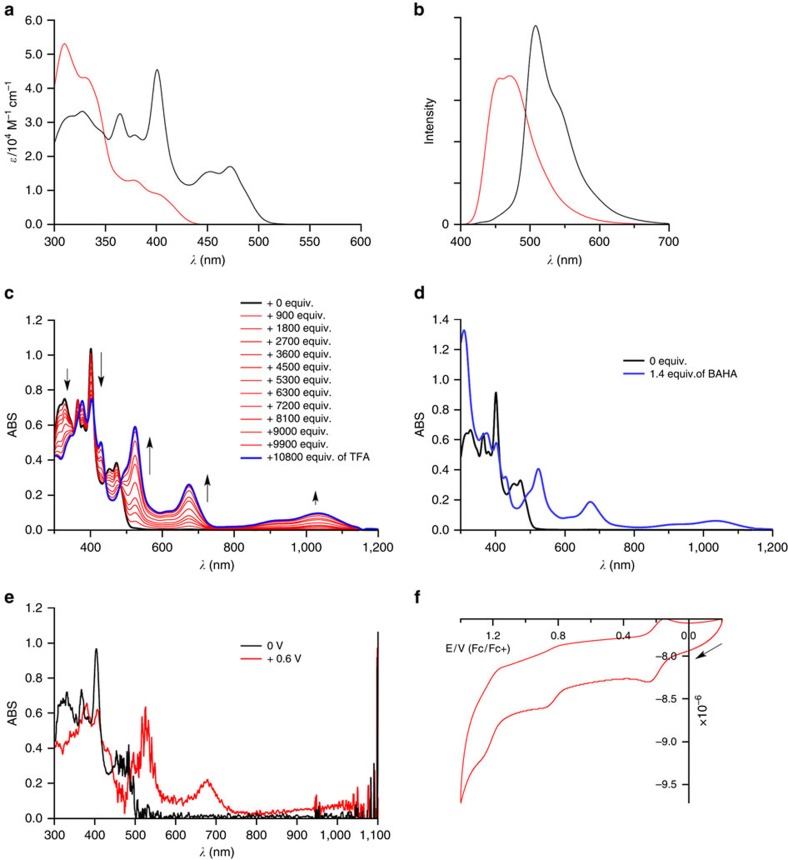
Physical properties of 5. (**a**) Ultraviolet–visible absorption spectra in CH_2_Cl_2_ and (**b**) emission spectra of **3** (red) and **5** (black) in CH_2_Cl_2_ (concentration: 7.6 × 10^−7^ M). (**c**) Spectral changes in absorption spectra of **5** on the addition of TFA into a dichloromethane solution of **5**. (**d**) Absorption spectra of **5** in CH_2_Cl_2_ before and after the addition of 1.4 equiv. of BAHA. (**e**) Spectroelectrochemical analysis of **5** in CH_2_Cl_2_. (**f**) Cyclic voltammogram of **5** measured in CH_2_Cl_2_ with tetra-*n*-butylammonium hexafluorophosphate as the electrolyte.

Figure 3 shows ultraviolet–visible absorption and emission spectra of **3** and **5** in CH_2_Cl_2_. The lowest energy bands shifted to the low-energy region as the degree of fusion increased. All compounds exhibited fluorescence in the visible region (Fig. 3b). The emission quantum yield of **5** was 17%, which is the highest among buckybowls^35^. The Stokes shifts of **3** (2,800 cm^−1^) and **5** (1,500 cm^−1^) were relatively larger than that of a planar molecule. This reflects their excited state dynamics, owing to their distorted characteristics.

The electronic structures of **3** and **5** were further investigated by an electrochemical analysis (Supplementary Fig. 22 and Supplementary Table 2). Reversible oxidation waves were observed for all compounds. The first oxidation potentials were lowered in the order of **2**>**3**>**5**, indicating effective electron donation from the nitrogen atom to the entire *π* system.

We then examined the protonation behaviour of **5**, because **5** was expected to have higher basicity than planar amines. The addition of trifluoroacetic acid (TFA) to a dichloromethane solution of **5** induced a dramatic change in its absorption spectrum (Fig. 3c). Interestingly, the same change was observed on the addition of a one-electron oxidant, tris(4-bromophenyl)aminium hexachloroantimonate (BAHA) (Fig. 3d). We also monitored the electro-oxidative absorption spectrum of **5** in CH_2_Cl_2_, which exhibited essentially the same change (Fig. 3e). These facts strongly indicate that the addition of TFA resulted in the generation of the radical cation species rather than simple protonation. The formation of the radical cation was confirmed by electron spin resonance (ESR) measurements (Supplementary Fig. 26). The solution of **5** in the presence of TFA exhibited a distinct signal at *g*=2.002, as was the case of the oxidation of **5** with BAHA. The conversion of **5** to the radical cation was almost quantitative under air atmosphere but was substantially lower under argon (Supplementary Fig. 27). The radical cation generation is likely due to electron transfer between **5** and protonated **5** involving air oxidation^36^. The facile generation of the radical cation from **5** would allow the investigation of the effect of oxidative doping on solid-state properties. This phenomenon also implies that nitrogen-doped electron-rich nanocarbons may undergo a similar radical cation generation by protonation.

### Association behaviour of **5** with C_60_

**Figure 4:**
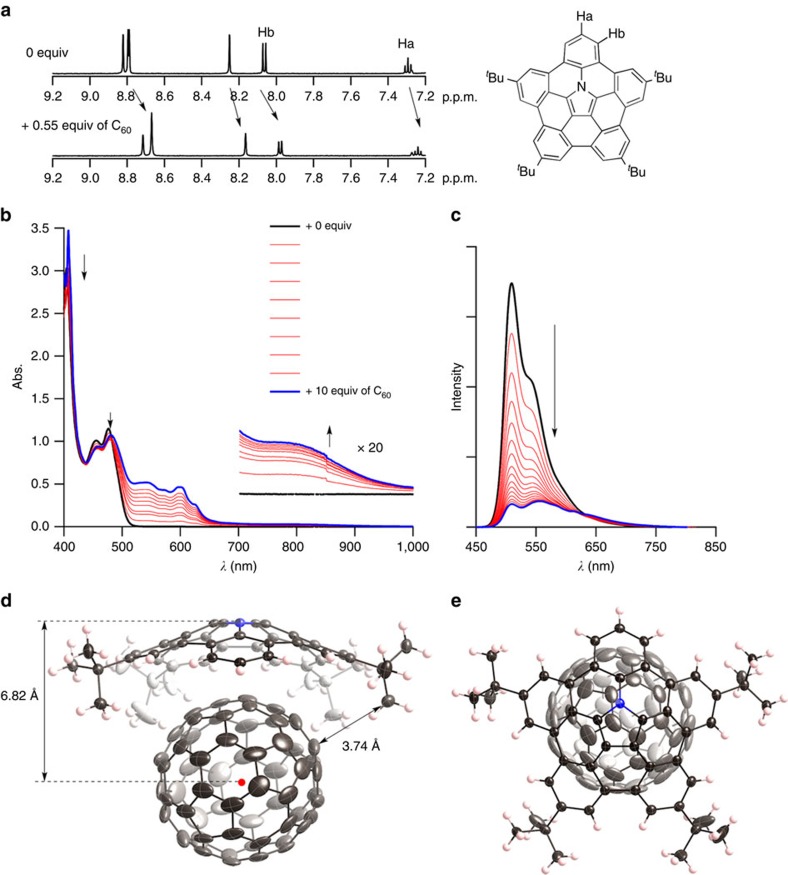
C_60_ binding behaviour of 5 in solution and solid. (**a**) ^1^H NMR spectra before (top) and after (bottom) addition of 0.55 equiv. of C_60_ into a toluene-*d*_8_ solution of **5**. (**b**) Ultraviolet–visible absorption spectra of addition of 0–10 equiv. of C_60_ into a 1,2-dichlorobenzene solution of **5**. (**c**) Fluorescence spectra of addition from 0–150 equiv. of C_60_ into a 1,2-dichlorobenzene solution of **5**. (**d**) Side view and (**e**) top view of X-ray crystal structure of **5⊃C**_**60**_. Thermal ellipsoids are scaled at 50% probability level. Solvent molecules (toluene) in the crystal structure of **5⊃C**_**60**_ were omitted for clarity.

The effect of nitrogen also appeared in the association behaviour of **5** with C_60_. For hydrocarbon buckybowls, their association constants with C_60_ were very low to be measured^37^,^38^,^39^,^40^. The incorporation of electron-rich nitrogen in buckybowls should enable tighter binding with electron-deficient fullerenes. The electrochemical analysis revealed its much lower oxidation potential (0.20 V) when compared with corannulene (1.57 V) (Fig. 3f)^41^. The addition of C_60_ into an 1,2-dichlorobenzene solution of **5** induced a change in the ultraviolet–visible absorption and emission spectra (Fig. 4b,c). In particular, the appearance of broad absorption bands in the near-infrared region suggests intermolecular charge-transfer interactions between **5** and C_60_. The association behaviour was also monitored by ^1^H NMR analysis. On the addition of C_60_ into a toluene-*d*_8_ solution of **5**, all aromatic proton signals were upfield shifted (Fig. 4a). This indicates that **5** and C_60_ interacted in a convex–concave manner. This was revealed by the X-ray crystallographic analysis and showed that C_60_ was located above the centre of **5** (Fig. 4d,e). The penetration depth of C_60_ into **5** measured from the centroid of the pyrrole ring to the centroid of C_60_ is 6.82 Å, and that measured from the shortest distance from the concave surface of **5** to a C_60_ surface is 3.29 Å, whereas the depths of C_60_ into the corannulene/C_60_ complex are 6.94 and 3.75 Å. The short distance between **5** and C_60_ indicates the presence of attractive interactions between them. Judging from the relatively long distance (>3.74 Å), the CH–*π* interaction between *tert*-butyl groups and C_60_ was not essential. The binding constant was determined to be 3,800 M^−1^ by titration with absorption and fluorescence spectra (Supplementary Figs 23–25). This value is approximately three times larger than that of perthiolated corannulene. The existence of intermolecular charge-transfer interactions between **5** and C_60_ was indicated by the quenching behaviour of the emission on the addition of C_60_ to a 1,2-dichlorobenzene solution of **5** (Fig. 4c). The DFT optimization of **5⊃C**_**60**_ afforded nearly the same structure as the crystal structure. The highest occupied molecular orbital was spread over the entire surface of **5**, whereas the lowest unoccupied molecular orbital was delocalized on C_60_ (Supplementary Fig. 29). In addition, oscillator strengths of the absorption of **5⊃C**_**60**_ were simulated by the time-dependent DFT method (Supplementary Fig. 30). The broad lowest-energy band in the near-infrared region was assigned as the highest occupied molecular orbital–lowest unoccupied molecular orbital transition. These results supported the conclusion that an intermolecular charge-transfer interaction exists between **5** and C_60_.

**Figure 5:**
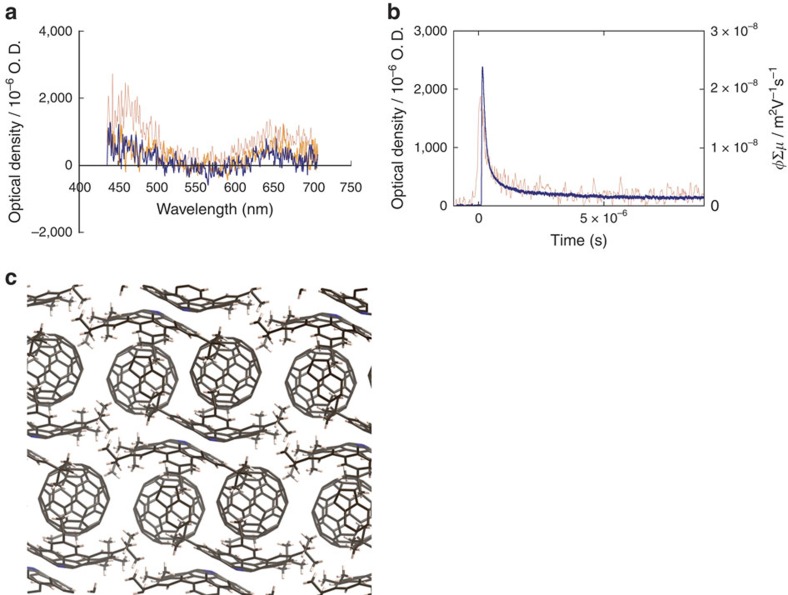
Charge-carrier mobility of 5⊃C_60_. (**a**) Transient absorption spectra of **5⊃C**_**60**_ on exposure to 355 nm laser pulses at 3.8 × 10^16^ photons per cm^2^. Spectra wire observed immediately after pulse exposure (red), 2 ms (orange) and 7 ms (blue) after pulse exposure. All spectra were recorded at room temperature under air-saturated atmosphere. (**b**) Kinetic traces of a photoconductivity transient (blue) recorded by FP-TRMC measurements and transient optical absorption at 650 nm for **5⊃C**_**60**_ on exposure to 355 nm laser pulses at 9.1 × 10^15^ photons per cm^2^ (conductivity) and 3.8 × 10^16^ photons per cm^2^ (optical). (**c**) Side view of crystal packing of **5⊃C**_**60**_. Solvent molecules (toluene) were omitted for clarity.

Finally, we investigated the effect of the association with C_60_ of **5** on the charge-carrier mobility of **5** by flash-photolysis time-resolved microwave conductivity (FP-TRMC) measurements^42^. The maximum transient conductivity (*φ*Σ*μ*) of **5** was measured to be 1.5 × 10^−5^ cm^2^ V^−1^ s^−1^ (Supplementary Fig. 28). For the co-crystal of **5⊃C**_**60**_, the mobility was enhanced to 2.4 × 10^−4^ cm^2^ V^−1^ s^−1^ (Fig. 5a,b). The charge-carrier generation efficiency (*φ*) was determined to be 4.4 × 10^−3^ by the transient absorption spectroscopy measurement. Accordingly, the local charge mobility of **5⊃C**_**60**_ was 0.17 cm^2^ V^−1^ s^−1^. Such a large mobility of **5⊃C**_**60**_ should originate from effective charge separation caused by an electronic interaction between **5** and C_60_. Furthermore, the alignment of **5** and C_60_ in the co-crystal may contribute to mobility enhancement. Both C_60_ and **5** construct one-dimensional chain alignments in the co-crystal (Fig. 5c).

## Discussion

In summary, we have achieved the synthesis of a nitrogen-embedded buckybowl under mild conditions. The total yield of azabuckybowl **5** was 11% from 9-bromophenanthrene. We also found that the protonation of **5** resulted in the efficient generation of radical cation species. The nitrogen-embedded buckybowl was sufficiently electron-rich to assemble tightly with C_60_ in solution and solid states. The molecular assembly of **5** with C_60_ exhibited a significantly high charge mobility (0.17 cm^2^ V^−1^ s^−1^). The nitrogen-embedded buckybowl can be a novel molecular entity in the field of curved *π* systems as fullerene hosts, anisotropic *π* donors and precursors to nitrogen-containing nanocarbon materials.

## Method

### Materials and characterization

^1^H NMR (500 MHz) and ^13^C NMR (126 MHz) spectra were recorded using a Bruker AVANCE III HD spectrometer. Chemical shifts were reported at the delta scale in p.p.m. relative to CHCl_3_ (*δ*=7.260 p.p.m.), CH_2_Cl_2_ (*δ*=5.320 p.p.m.), toluene-*d*_8_ (*δ*=7.000 p.p.m.), acetone-*d*_*6*_ (*δ*=2.05 p.p.m.) and 1,2-dichlorobenzene-*d*_4_ (*δ*=6.930 p.p.m.) for ^1^H NMR and CDCl_3_ (*δ*=77.0 p.p.m.) for ^13^C NMR. ^1^H and ^13^C NMR spectra are provided for all compounds; see Supplementary Figs 1–17. Ultraviolet–visible–near infrared absorption spectra were recorded using a Shimadzu UV-2550 or JASCO V670 spectrometer. Emission spectra were recorded using a JASCO FP-6500 spectrometer and absolute fluorescence quantum yields were measured by the photon-counting method using an integration sphere. Mass spectra were recorded using a Bruker microTOF by electrospray ionization (ESI) methods. Unless otherwise noted, materials obtained from commercial suppliers were used without further purification.

### Synthesis of 3,6-Di-*tert*-butyl-9-bromophenanthrene

3,6-Di-*tert*-butylphenanthrene (0.540 g, 1.86 mmol) was dissolved in CCl_4_ (11 ml) in a two-necked flask equipped with a dropping funnel. Br_2_ (0.10 ml, 1.95 mmol) and CCl_4_ (11 ml) were added into the dropping funnel. The solution was heated to 50 °C and then the bromine solution was added slowly over 1 h. After the addition was completed, the mixture was stirred for additional 30 min. The reaction mixture was cooled to room temperature and then quenched with aqueous Na_2_S_2_O_3_. The resulting mixture was extracted with CH_2_Cl_2_ and the organic layer was washed with aqueous Na_2_S_2_O_3_, dried over Na_2_SO_4_ and concentrated *in vacuo*. Purification by silica-gel column chromatography (cyclohexane as eluent) afforded the title compound (0.645 g, 1.75 mmol) in 94% yield as a white solid. ^1^H NMR (500 MHz) (CDCl_3_): *δ*=8.70 (d, *J*=1.5 Hz, 1H), 8.66 (s, 1H), 8.30 (d, *J*=8.5 Hz, 1H), 8.02 (s,1H), 7.77 (dd, *J*_1_=8.5 Hz, *J*_2_=1.5 Hz, 1H), 7.74 (d, *J*=8.5 Hz, 1H), 7.68 (dd, *J*_1_=8.5 Hz, *J*_2_=1.5 Hz, 1H), 1.53 (s, 9H), 1.52 (s, 9H) p.p.m.; ^13^C NMR (126 MHz) (CDCl_3_): *δ*=150.0, 149.6, 131.1, 130.4, 129.5, 129.3, 128.5, 127.8, 127.5, 125.6, 125.4, 120.7, 118.2, 118.1, 35.25, 35.14, 31.42 p.p.m.; high-resolution atmospheric pressure chemical ionization–MS (APCI–MS): *m/z*=368.1124, calcd for (C_22_H_25_Br)^+^=368.1134 [*M*^+^].

### Synthesis of compound **1**

A Schlenk tube containing 3,6-di-*tert*-butyl-9-bromophenanthrene (0.200 g, 0.542 mmol), Cs_2_CO_3_ (0.265 g, 0.812 mmol), Pd_2_dba_3_·CHCl_3_ (28.0 mg, 27.1 μmol) and Xantphos (31.3 mg, 54.0 μmol) was flushed with N_2_ three times. To the tube, 2-chloroaniline (86 μl, 0.812 mmol) and dry 1,4-dioxane (2.0 ml) were added. The mixture was stirred for 46 h at 100 °C. The resulting mixture was cooled to room temperature, passed through a pad of Celite and concentrated *in vacuo*. Purification by silica-gel column chromatography (hexane/CH_2_Cl_2_) afforded **1** (0.158 g, 0.380 mmol) in 70% yield as a pale yellow solid. ^1^H NMR (500 MHz) (CDCl_3_): *δ*=8.76 (d, *J*=2.0 Hz, 1H), 8.68 (d, *J*=1.0 Hz, 1H), 8.04 (d, *J*=8.5 Hz, 1H), 7.74 (d, *J*=8.5 Hz, 1H), 7.69 (dd, *J*_1_=8.5 Hz, *J*_2_=2.0 Hz, 1H), 7.66 (dd, *J*_1_=8.5 Hz, *J*_2_=1.5 Hz, 1H), 7.61 (s, 1H), 7.41 (dd, *J*_1_=8.0 Hz, *J*_2_=1.5 Hz, 1H), 7.04 (ddd, *J*_1_=8.5 Hz, *J*_2_=7.0 Hz, *J*_3_=1.5 Hz, 1H), 6.88 (dd, *J*_1_=8.5 Hz, *J*_2_=1.5 Hz, 1H), 6.77 (ddd, *J*_1_=*J*_2_=8.0 Hz, *J*_3_=1.5 Hz, 1H), 6.36 (s, 1H), 1.53 (s, 9H), 1.52 (s, 9H) p.p.m.; ^13^C NMR (126 MHz) (CDCl_3_): *δ*=149.6, 148.6, 142.4, 134.6, 131.4, 130.2, 129.5, 128.5, 127.7, 127.6, 127.1, 125.1, 125.0, 122.8, 120.5, 119.4, 118.9, 118.6, 117.9, 115.6, 35.18, 31.54, 31.48 p.p.m.; high-resolution APCI–MS: *m/z*=416.2141, calcd for (C_28_H_31_ClN)^+^=416.2140 [(*M+H*)^+^].

### Synthesis of compound **2**

A flask-containing compound **1** (0.100 g, 0.241 mmol) was flushed with N_2_ three times. To the flask, a dry and degassed toluene/CF_3_COOH (10 ml, 33 μl) solution was added. To the solution, a solution of DDQ (0.109 g, 0.482 mmol) in dry and degassed toluene/CF_3_COOH (10 ml, 33 μl) was added and the mixture was stirred for 1 h at room temperature. The reaction mixture was quenched with aqueous NaHCO_3_ and aqueous Na_2_S_2_O_3_, and extracted with CH_2_Cl_2_. The organic layer was washed with water, dried over Na_2_SO_4_ and concentrated *in vacuo*. Purification by silica-gel column chromatography (hexane/CH_2_Cl_2_) afforded compound **2** (79.6 mg, 0.113 mmol) in 94% yield as a pale yellow solid. ^1^H NMR (500 MHz) (CDCl_3_): *δ*=9.00 (d, *J*=8.5 Hz, 2H), 8.80 (d, *J*=1.5 Hz, 2H), 8.75 (d, *J*=1.5 Hz, 2H), 7.85 (dd, *J*_1_=8.5 Hz, *J*_2_=1.5 Hz, 1H), 7.69–7.77 (m, 4H), 7.62 (ddd, *J*_1_=*J*_2_=7.5 Hz, *J*_3_=1.5 Hz, 1H), 7.32 (dd, *J*_1_=9.0 Hz, *J*_2_=1.5 Hz, 2H), 7.08 (d, *J*=9.0 Hz, 2H), 1.58 (s, 18H), 1.48 (s, 18H) p.p.m.; ^13^C NMR (126 MHz) (CDCl_3_): *δ*=147.3, 146.8, 140.7, 135.7, 132.6, 132.2, 131.3, 131.1, 130.6, 128.9, 127.8, 126.6, 125.8, 124.4, 123.6, 121.7, 120.5, 119.7, 119.4, 116.5, 35.02, 34.91, 31.61, 31.40 p.p.m.; ultraviolet–visible (CH_2_Cl_2_): *λ*_max_ (*ɛ*[M^−1^cm^−1^])=342 (22,000), 359 (23,000), 376 (21,000) nm; high-resolution APCI–MS: *m/z*=702.3872, calcd for (C_50_H_53_ClN)^+^=702.3861 [(*M+H*)^+^].

### Synthesis of compound **3**

A Schlenk tube containing compound **2** (30.1 mg, 42.8 μmol), K_2_CO_3_ (35.5 mg, 0.257 mmol), Pd(OAc)_2_ (9.56 mg, 42.6 μmol) and PCy_3_·HBF_4_ (31.5 mg, 85.5 μmol) was flushed with N_2_ three times. To the tube, dry and degassed DMA (1.5 ml) was added. The mixture was stirred for 43 h at 130 °C. The resulting mixture was cooled to room temperature, passed through a pad of Celite and concentrated *in vacuo*. Purification by silica-gel column chromatography (hexane/CH_2_Cl_2_) afforded compound **3** (17.8 mg, 26.8 μmol) in 63% yield as a yellow solid. ^1^H NMR (500 MHz) (CDCl_3_): *δ*=9.15 (d, *J*=9.0 Hz, 1H), 9.13 (d, *J*=10 Hz, 1H) 8.92 (d, *J*=1.0 Hz, 1H), 8.89 (s, 1H), 8.86 (d, *J*=0.5 Hz, 1H), 8.83 (d, *J*=0.5 Hz, 1H), 8.61 (dd, *J*_1_=8.0 Hz, *J*_2_=1.5 Hz, 1H), 8.59 (s, 1H), 8.51 (d, *J*=8.5 Hz, 1H), 8.40 (d, *J*=8.0 Hz, 1H), 7.88 (d, *J*=8.5 Hz, 1H), 7.84 (d, *J*=8.5 Hz, 1H), 7.66 (dd, *J*_1_=8.5 Hz, *J*_2_=1.5 Hz, 1H), 7.50–7.56 (m, 2H), 1.71 (s, 9H), 1.64 (s, 9H), 1.63 (s, 9H), 1.60 (s, 9H) p.p.m.; ^13^C NMR (126 MHz) (CDCl_3_): *δ*=148.9, 147.7, 147.7, 146.5, 135.2, 132.2, 129.3, 129.1, 129.0, 128.9, 127.5, 127.3, 127.2, 126.9, 126.6, 126.1, 125.4, 124.7, 124.6, 124.2, 124.2, 124.0, 123.4, 123.1, 122.8, 121.9, 120.1, 119.7, 119.6, 119.5, 118.4, 118.1, 116.0, 110.8, 35.84, 35.12, 35.10, 35.01, 32.00, 31.65, 31.64, 31.56 p.p.m.; ultraviolet–visible (CH_2_Cl_2_): *λ*_max_ (*ɛ*[M^−1^ cm^−1^])=310 (53,000), 330 (43,000), 378 (13,000) and 404 (8,700) nm; fluorescence (CH_2_Cl_2_, *λ*_ex_=378 nm): *λ*_em_=456 and 471 nm (*Φ*_f_=0.18); high-resolution APCI–MS: *m/z*=666.4088, calcd for (C_50_H_52_N)^+^=666.4094 [(*M+H*)^+^].

### Synthesis of compound **4**

Compound **3** (40.1 mg, 60.3 μmol) was dissolved in CCl_4_ (6.0 ml) in a two-necked flask equipped with a dropping funnel. A solution of Br_2_ (0.10 ml, 2.0 mmol) in CCl_4_ (3.0 ml) was added to the dropping funnel. The mixture was heated to 70 °C and then the bromine solution was added slowly over 15 min. After the addition was complete, the mixture was stirred for an additional 12.5 h. The reaction mixture was cooled to room temperature and then quenched with aqueous Na_2_S_2_O_3_. The resulting mixture was extracted with CH_2_Cl_2_ and the organic layer was washed with aqueous Na_2_S_2_O_3_, dried over Na_2_SO_4_ and concentrated *in vacuo*. Purification by silica-gel column chromatography (hexane only) afforded compound **4** (30.3 mg, 33.6 μmol) in 56% yield as a yellow solid. ^1^H NMR (500 MHz) (CDCl_3_): *δ*=8.83 (s, 1H), 8.80 (s,1H), 8.75 (s, 1H), 8.59 (d, *J*=1.5 Hz, 1H), 8.58 (s, 1H), 8.47 (s, 1H), 8.41 (d, *J*=8.5 Hz, 1H), 8.05 (s, 1H), 7.96 (d, *J*=9.0 Hz, 1H), 7.91 (s, 1H), 7.68 (dd, *J*_1_=7.8 Hz, *J*_2_=1.5 Hz, 1H), 7.49 (dd, *J*_1_=9.0 Hz, *J*_2_=2.0 Hz, 1H), 1.66 (s, 9H), 1.60 (s, 9H), 1.59 (s, 9H), 1.52 (s, 9H) p.p.m.; ^13^C NMR (126 MHz) (CDCl_3_): *δ*=149.7, 149.0, 147.9, 147.9, 135.7, 132.3, 132.1, 131.0, 130.6, 130.2, 129.9, 129.4, 129.4, 127.8, 127.6, 127.1, 126.8, 125.5, 124.9, 124.6, 124.3, 124.2, 123.4, 121.0, 120.3, 119.9, 119.7, 118.9, 118.8, 118.8, 118.1, 117.4, 117.1, 108.8, 35.91, 35.13, 35.05, 34.99, 31.93, 31.54, 31.51 p.p.m.; high-resolution APCI–MS: *m/z*=900.1403, calcd for (C_50_H_49_Br_3_N)^+^=900.1410 [(*M+H*)^+^].

### Synthesis of compound **5**

A Schlenk tube containing compound **4** (20.3 mg, 22.5 μmol), K_2_CO_3_ (24.8 mg, 0.180 mmol), Pd(OAc)_2_ (10.7 mg, 47.7 μmol) and PCy_3_·HBF_4_ (33.2 mg, 90.0 μmol) was flushed with N_2_ three times. To the tube, dry and degassed DMA (2.6 ml) was added. The mixture was stirred for 16 h at 130 °C. The resulting mixture was cooled to room temperature and extracted with ethyl acetate. The organic layer was washed with water, dried over Na_2_SO_4_ and concentrated *in vacuo*. Purification by silica-gel column chromatography (hexane only) afforded compound **5** (6.81 mg, 10.3 μmol) in 46% yield as a yellow solid. ^1^H NMR (500 MHz) (CDCl_3_): *δ*=8.61 (s, 2H), 8.54 (s, 2H), 8.52 (s, 2H), 8.23–8.24 (m, 4H), 7.50 (t, *J*=8.0 Hz, 1H), 1.63 (s, 18H), 1.60 (s, 18H) p.p.m.; ^13^C NMR (126 MHz) (CDCl_3_): *δ*=148.8, 147.6, 140.1, 135.3, 132.5, 131.1, 130.0, 129.0, 128.6, 127.7, 126.1, 123.4, 123.0, 122.5, 120.5, 120.0, 119.4, 117.9, 35.92, 35.84, 32.30, 32.17 p.p.m.; ultraviolet–visible (CH_2_Cl_2_): *λ*_max_ (*ɛ*[M^−1^cm^−1^])=400 (35,000), 453 (12,000), 472 (13,000) nm; fluorescence (CH_2_Cl_2_, *λ*_ex_=400 nm): *λ*_em_=508 and 542 nm (*Φ*_f_=0.17); high-resolution APCI–MS: *m/z*=662.3748, calcd for (C_50_H_48_N)^+^=662.3781 [(*M*+*H*)^+^].

### Synthesis of compound **6**

A Schlenk tube containing compound **5** (30.5 mg, 46.0 μmol), bis(pinacolato)diboron (117 mg, 0.461 mmol), [Ir(OMe)(cod)]_2_ (30.5 mg, 46.0 μmol) and 4,4'-di-*tert*-butyl-2,2'-bipyridyl (25.1 mg, 93.4 μmol) was flushed with N_2_ three times. To the tube, dry and degassed octane (1.5 ml) was added. The mixture was stirred for 10.5 h at 110 °C. The resulting mixture was cooled to room temperature and concentrated *in vacuo*. Purification by silica-gel column chromatography afforded compound **6** (29.0 mg, 36.8 μmol) in 80% yield as a yellow solid. ^1^H NMR (500 MHz) (CDCl_3_): *δ*=8.68 (s,2H), 8.62 (s, 2H), 8.55 (s, 2H), 8.52 (s, 2H), 8.34 (s, 2H), 1.64 (s, 18H), 1.64 (s, 18H), 1.49 (s, 12H) p.p.m.; ^13^C NMR (126 MHz) (CDCl_3_): *δ*=148.9, 147.7, 140.0, 137.1, 132.6, 130.9, 130.1, 128.9, 128.5, 127.7, 125.4, 123.0, 120.4, 120.0, 119.4, 118.4, 84.21, 36.01, 35.84, 32.29, 32.24, 24.99 p.p.m.; high-resolution ESI–MS: *m/z*=787.4571, calcd for (C_56_H_58_BNO_2_)^+^=787.4564 [(*M*)^+^].

### Synthesis of compound **7**

A Schlenk tube containing compound **6** (9.48 mg, 12.0 μmol), PdCl_2_dppf·CH_2_Cl_2_ (4.97 mg, 6.09 μmol) and Cs_2_CO_3_ (9.80 mg, 30.1 μmol) was flushed with N_2_ three times. To the tube, 2,4,6-triisopropylbromobenzene (56.2 mg, 0.199 mmol) and dry and degassed 1,4-dioxane (1.0 ml) were added. The mixture was stirred for 13 h at 100 °C. The resulting mixture was cooled to room temperature and concentrated *in vacuo*. Purification by silica-gel column chromatography afforded compound **7** (5.75 mg, 6.66 μmol) in 55% yield as a yellow solid. ^1^H NMR (500 MHz) (acetone-*d*_*6*_): *δ*=8.85 (s, 2H), 8.79 (s, 2H), 8.79 (s, 2H), 8.65 (d, *J*=1.0 Hz, 2H), 8.36 (s, 2H), 7.25 (d, *J*=1.5 Hz, 1H), 7.14 (d, J=1.5 Hz, 1H), 3.13 (sext, *J*=7.0 Hz, 1H), 3.01 (sext, *J*=7.0 Hz, 1H), 2.37 (sext, *J*=7.0 Hz, 1H), 1.62 (s, 18H), 1.57 (s, 18H), 1.35 (d, *J*=7.0 Hz, 6H), 1.31 (d, *J*=7.0 Hz, 6H), 0.88 (d, *J*=7.0 Hz, 6H) p.p.m.; ^1^H NMR (500 MHz) (1,2-dichlorobenzene-*d*_4_): *δ*=8.75 (s, 2H), 8.74 (s, 2H), 8.72 (s, 2H), 8.25 (s, 2H), 8.21 (s, 2H), 7.35 (s, 1H), 7.28 (s, 1H), 3.35 (sext, *J*=7.5 Hz, 1H), 3.02 (sext, *J*=7.0 Hz, 1H), 2.85 (sext, *J*=7.0 Hz, 1H), 1.66 (s, 18H), 1.50 (s, 18H), 1.41 (d, *J*=7.0 Hz, 6H), 1.37 (d, 6H), 1.11 (d, *J*=7.0 Hz, 6H) p.p.m.; ^13^C NMR (126 MHz) (CDCl_3_): *δ*=148.9, 148.5, 147.6, 147.1, 147.0, 140.4, 137.3, 135.9, 134.3, 132.5, 131.2, 130.1, 129.3, 128.5, 127.9, 125.9, 123.9, 123.3, 121.1, 120.7, 120.5, 119.9, 119.3, 117.9, 35.89, 35.84, 34.38, 32.31, 32.10, 30.66, 30.10, 24.36, 24.14, 24.06 p.p.m.; high-resolution ESI–MS: *m/z*=863.5438, calcd for (C_65_H_69_N)^+^=863.5425 [(*M*)^+^].

### X-ray diffraction analysis

X-ray data were obtained using a Bruker D8 QUEST X-ray diffractometer with an IμS microfocus X-ray source and a large-area (10 cm × 10 cm) CMOS detector (Photon 100) for **3** and **4**, and using a Rigaku CCD diffractometer (Saturn 724 with MicroMax-007) with Varimax Mo optics using graphite monochromated Mo-Kα radiation (*λ*=0.71075 Å) for **5** and **5⊃C**_**60**_. For ORTEP structures of **3**, **4**, **5** and **5⊃C**_**60**_, see Supplementary Figs 18–21. Crystallographic details are given in CIF files (Supplementary Data 1–4). A fine crystal of **5** for the X-ray diffraction analysis was obtained by the vapour diffusion of methanol into its *o*-xylene solution. For the X-ray crystal structure of **5⊃C**_**60**_, a fine crystal for the X-ray diffraction analysis was obtained by the vapour diffusion of methanol into a toluene solution with a 1:1 mixture of **5** and C_60_. The molecule C_60_ was significantly disordered and refined as two disordered rigid bodies by restraining with DFIX, DANG, DELU and SIMU commands as generally used for the refinement of C_60_. The toluene solvent molecules were assigned as two disordered units by using minus part number, because it was located at the special position (Supplementary Fig. 21). The resolution and data were sufficiently suitable to determine the binding manner in the crystal (*R*_int_=0.0252, 22,169 total reflections and 11,448 unique reflections were observed). The detailed crystallographic data for all compounds are listed in Supplementary Table 1.

### Electrochemical analysis

The cyclic voltammogram and differential-pulse voltammogram of **5** were recorded using an ALS electrochemical analyser 612C. Measurements were performed in freshly distilled dichloromethane with tetrabutylammonium hexafluorophosphate as the electrolyte. A three-electrode system was used. The system consisted of a platinum working electrode, a platinum wire and Ag/AgClO_4_ as the reference electrode. The scan rate was 100 mVs^−1^. The measurement was performed under nitrogen atmosphere. All potentials are referenced to the potential of ferrocene/ferrocenium cation couple. The data are listed in Supplementary Table 2. The electro-oxidative absorption of **5** was recorded under argon atmosphere with a BAS SEC-F spectroelectrochemical flow cell kit equipped with a DH-2000-BAL as the ultraviolet–visible–near infrared light source and an HR4000CG–ultraviolet–near infrared spectrometer.

### Determination of binding constant

The binding constant (*K*_a_) of C_60_ with compound **5** was determined by ultraviolet–visible absorption and emission spectral analysis on the titration of C_60_ into the 1,2-dichlorobenzene solution of **5**. The fitting was performed with the correlation between the change of absorbance or fluorescence intensity (*ΔX*) at 700 and 508 nm, and the initial concentration of the guest ([G]_0_) using the equation as follows:


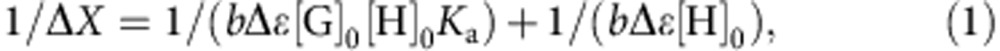


where *Δɛ* is the gap of molar coefficients between guest and complex, and [H]_0_ is the initial concentration of the host (Supplementary Figs 23 and 24). The estimated *K*_a_ values by ultraviolet–visible spectral analysis were 3.9 × 10^3^ M^−1^ for the first attempt and 3.7 × 10^3^ M^−1^ for the second attempt. The *K*_a_ was also estimated by the emission spectral analysis to be 3.8 × 10^3^ M^−1^. The average *K*_a_ is 3.8 × 10^3^ M^−1^.

### ESR measurement

ESR spectra were recorded at room temperature using a Bruker E500 spectrometer with 2.6*φ* quartz sample tubes. A sample solution of **5** was prepared under air and the ESR tube was sealed. Other samples were prepared by the addition of the degassed solution of TFA and BAHA in CH_2_Cl_2_ to the solution of **5**.

### Time-resolved microwave conductivity measurement

Transient photoconductivity was measured by FP-TRMC^43^. A resonant cavity was used to obtain a high degree of sensitivity in the conductivity measurement. The resonant frequency and microwave power were set at ∼9.1 GHz and 3 mW, respectively, such that the electric field of the microwave was sufficiently small not to disturb the motion of charge carriers. The conductivity value is converted to the product of the quantum yield *φ* and the sum of charge-carrier mobilities *Σμ* by *φΣμ*=*Δσ* (*eI*_0_*F*_light_)^−1^, where *e*, *I*_0_, *F*_light_ and *Δσ* are the unit charge of a single electron, incident photon density of excitation laser (photons per m^2^), a correction (or filling) factor (m^−1^) and a transient photoconductivity, respectively. The sample was set at the highest electric field in a resonant cavity. FP-TRMC experiments were performed at room temperature. The measurements of **5** and **5⊃C**_**60**_ were performed for crystalline samples covered with a polyvinyl alcohol film on a quartz substrate.

### Theoretical calculations

All calculations were performed using the Gaussian 09 programme^44^. The geometry of **5**^+·^, in which all *tert*-butyl groups were replaced with hydrogen, was optimized by the DFT method using the B3LYP^45^,^46^ functional and the 6-31G(d) basis set. The geometry of **5⊃C**_**60**_ was optimized by Zhao's M06-2X functional^47^ and the 6-31G(d) basis set. The oscillator strengths of **5**^+·^ and **5⊃C**_**60**_ were calculated by the time-dependent DFT method at the B3LYP/6-31G(d) level (Supplementary Figs 30 and 31). For calculations of the bowl-to-bowl inversion energy, the ground and transition state geometries of **7** were optimized at the B3LYP/cc-pVDZ level. Zero-point energy and thermal energy corrections were conducted for the optimized structures. The calculation results are summarized in Supplementary Tables 3–7.

### Determination of bowl-to-bowl inversion energy by 2D EXSY measurement

The bowl-to-bowl inversion barrier of **7** was measured by 2D EXSY using the signals for methine protons of isopropyl groups at approximately *δ*=3.3 and 2.8 p.p.m. (Supplementary Fig. 32)^48^. 2D EXSY measurements were performed in 1,2-dichlorobenzene-*d*_4_ at 393 K with a phase-sensitive nuclear Overhauser effect spectroscopy pulse sequence. The mixing time was increased from 50 to 300 ms. The rate constant (*k*) was determined using equation as follows:


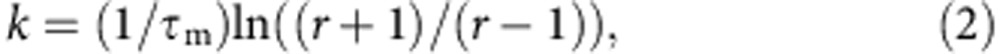


where *τ*_m_ is the mixing time and *r* is defined by the equation as follows:


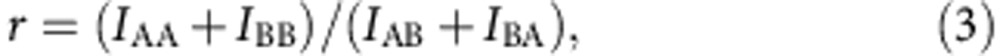


where *I*_AB_ and *I*_BA_ are the intensities of the cross-peaks between two exchangeable signals A and B, and *I*_AA_ and *I*_BB_ are the intensities of the diagonal signals (Supplementary Fig. 33). The free energy (*ΔG*^‡^) of the bowl-to-bowl inversion was finally obtained using the Eyring equation.

## Additional information

**Accession codes**: The X-ray crystallographic coordinates for structures reported in this study have been deposited at the Cambridge Crystallographic Data Centre (CCDC), under deposition numbers CCDC 1056760 (**3**), CCDC 1056759 (**4**), CCDC 1405427 (**5**) and CCDC 1406873 (**5⊃C**_**60**_). These data can be obtained free of charge from the CCDC via www.ccdc.cam.ac.uk/data_request/cif.

**How to cite this article:** Yokoi, H. *et al*. Nitrogen-embedded buckybowl and its assembly with C_60_. *Nat. Commun.* 6:8215 doi: 10.1038/ncomms9215 (2015).

## Supplementary Material

Supplementary Information Supplementary Figures 1-33 and Supplementary Tables 1-7

Supplementary Data 1 Crystal data for compound 3

Supplementary Data 2 Crystal data for compound 4

Supplementary Data 3 Crystal data for compound 5

Supplementary Data 4 Crystal data for 5-C60
