# Supplementary material for: Nitrogen-embedded buckybowl and its assembly with C60
Source: Nat Commun. 2015 Sep 4;6:8215. doi: 10.1038/ncomms9215 (PMC4569845; doi:10.1038/ncomms9215)
Supplement: Supplementary Information — Supplementary Figures 1-33 and Supplementary Tables 1-7 [file ncomms9215-s1.pdf]

* solvent and impurities

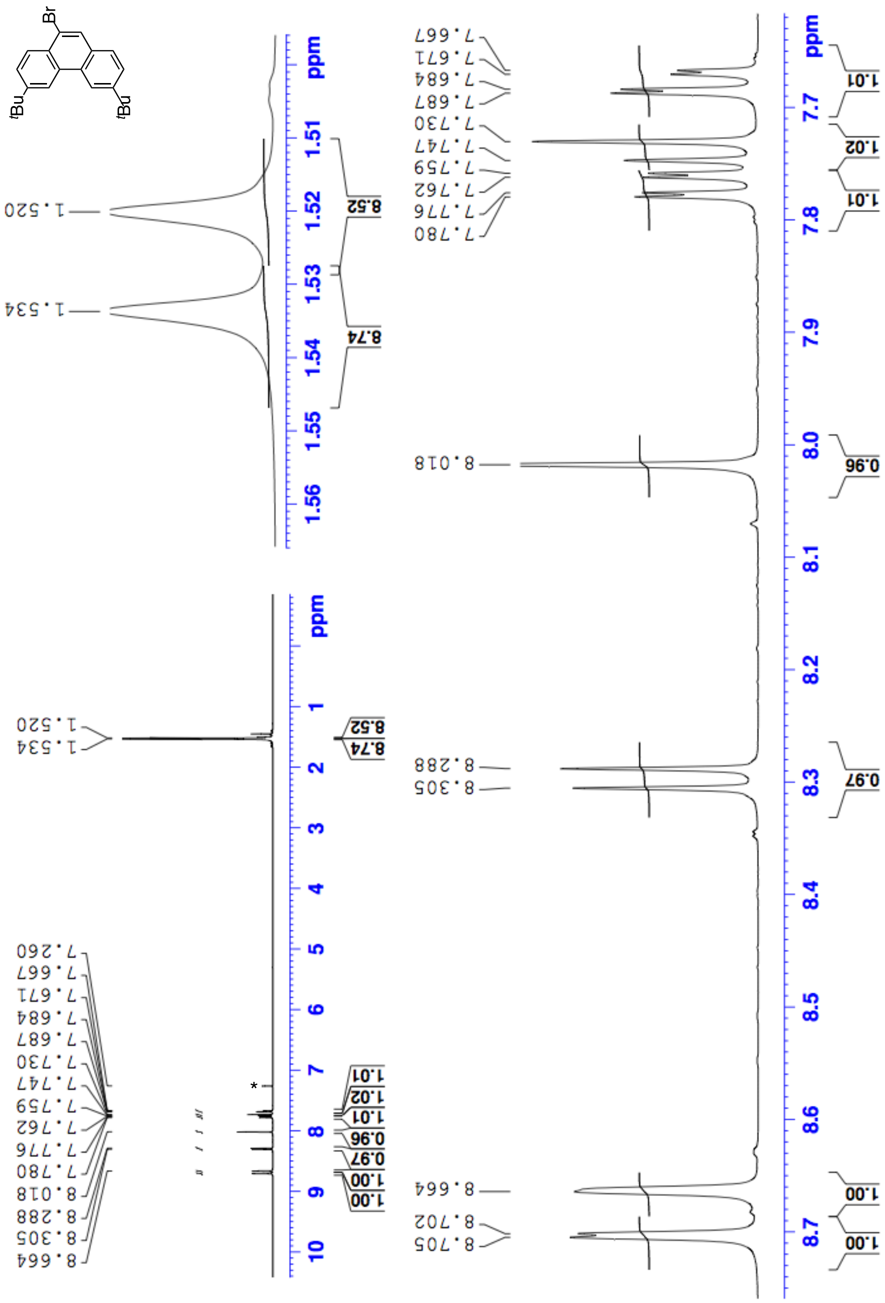

Supplementary Figure 1. ^{1}H NMR spectrum of 3,6-di-*tert*-butyl-9-bromophenenthrene in $CDCl_3$.

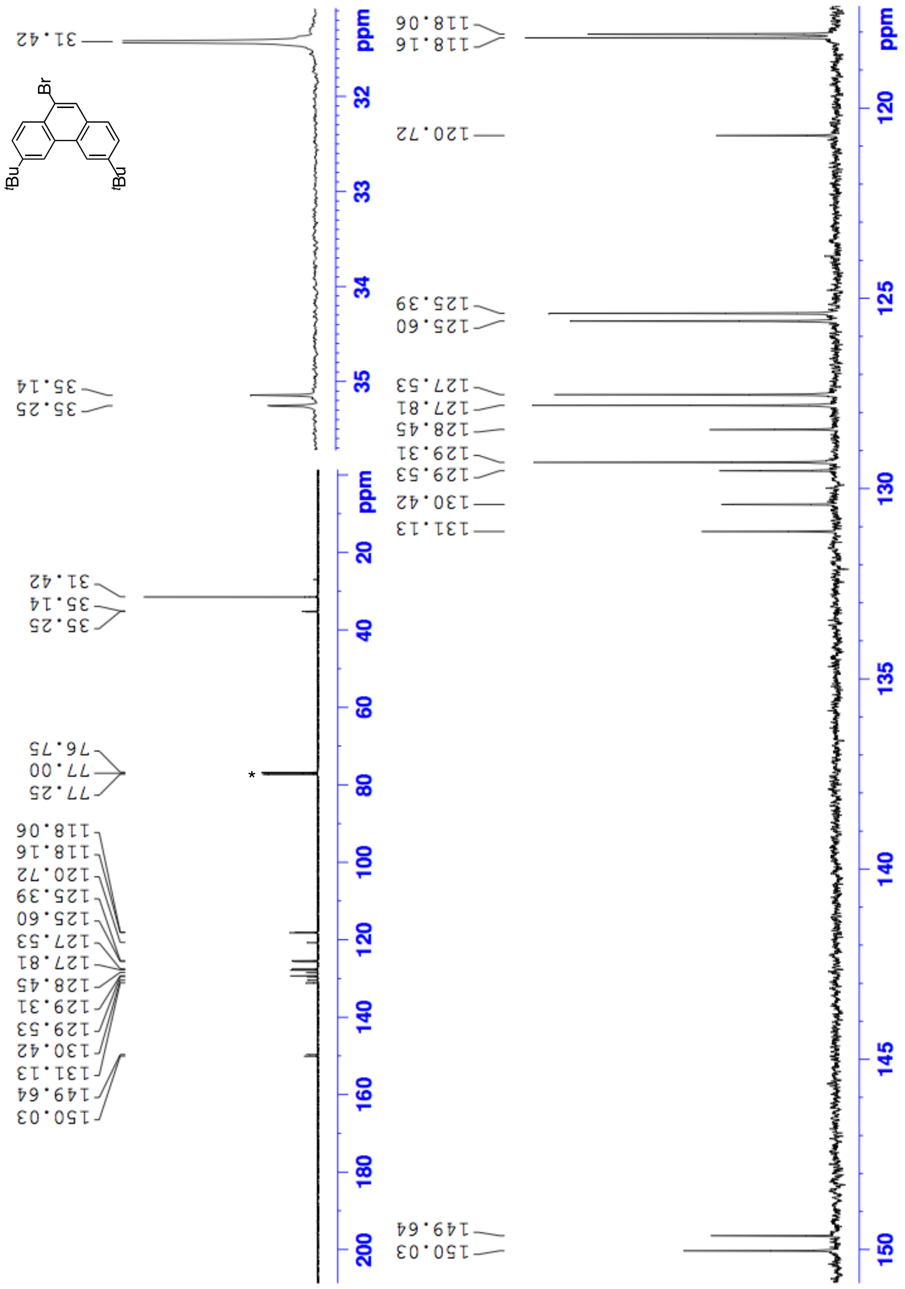

Supplementary Figure 2. ^{13}C NMR spectrum of 3,6-di-*tert*-butyl-9-bromophenenthrene

in $CDCl_3$.

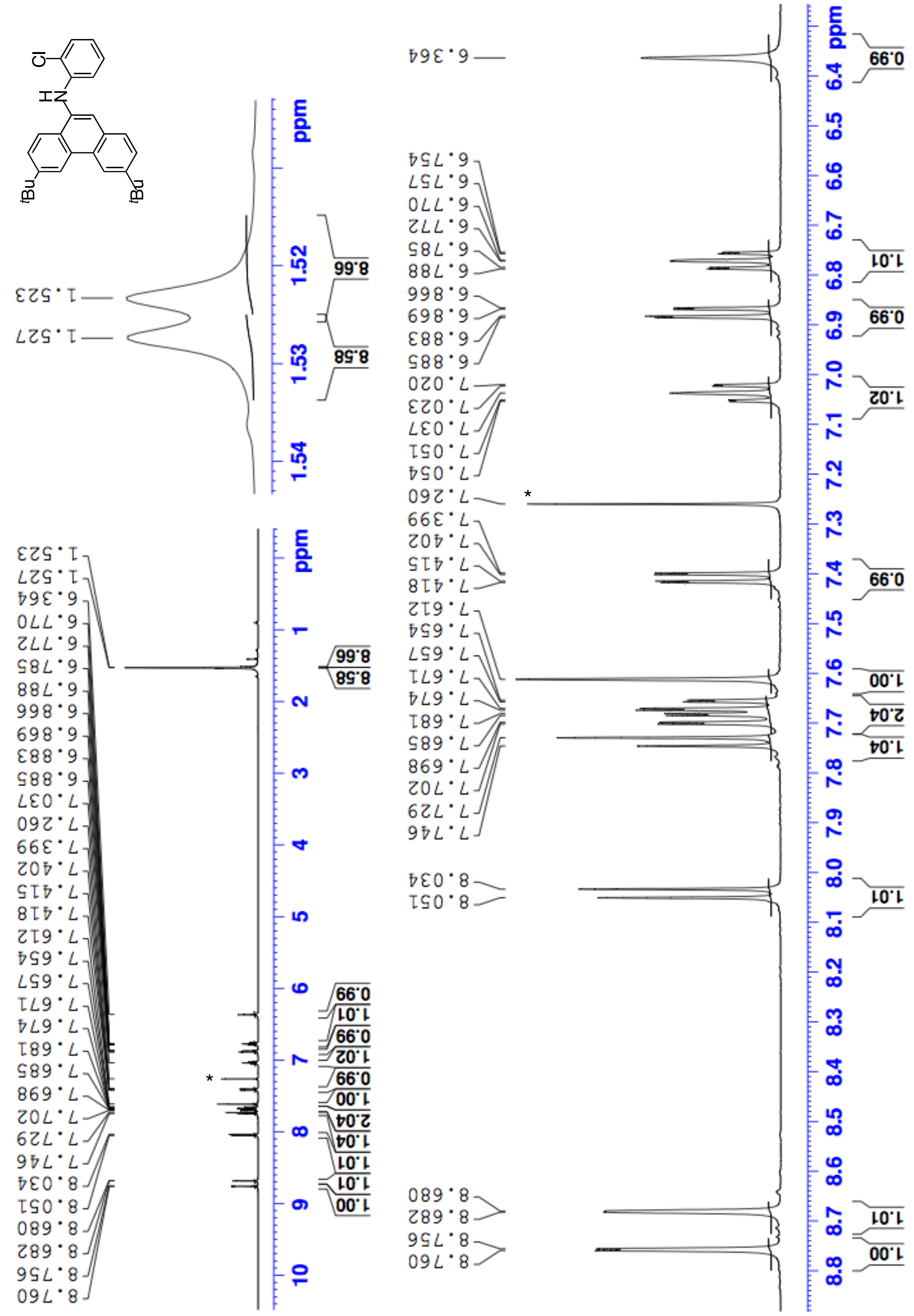

tBu
tBu
HN
Cl
1.523
1.527
6.364
6.770
6.772
6.785
6.788
6.866
6.869
6.883
6.885
7.037
7.260
7.399
7.402
7.415
7.418
7.612
7.654
7.657
7.671
7.674
7.681
7.685
7.698
7.702
7.729
7.746
8.034
8.051
8.680
8.682
8.756
8.760
ppm
8.66
8.58
0.99
1.01
0.99
1.02
0.99
1.00
2.04
1.04
1.01
1.01
1.00

Supplementary Figure 3. ¹H NMR spectrum of **1** in $CDCl_3$.

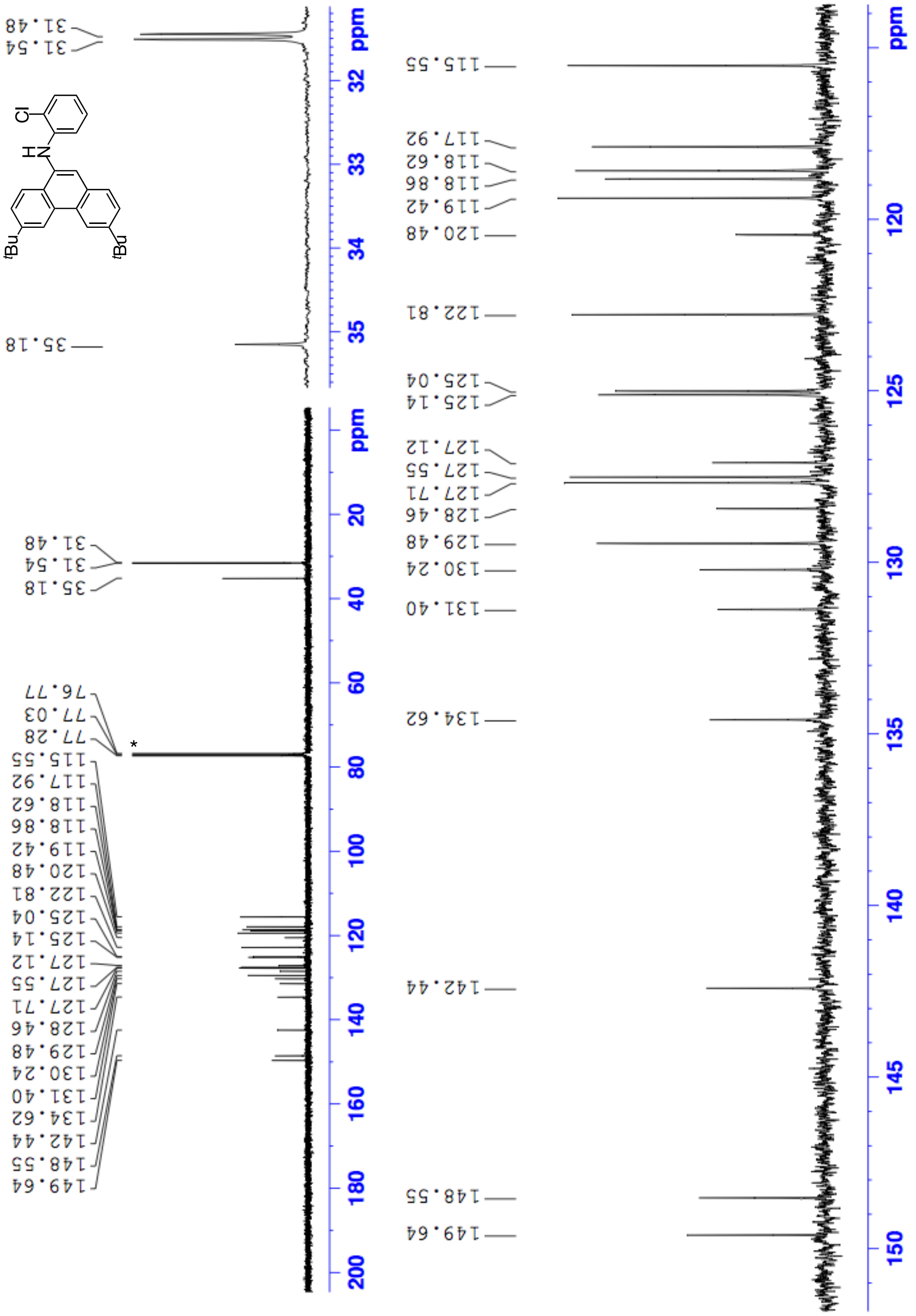

Supplementary Figure 4. ^{13}C NMR spectrum of **1** in $CDCl_3$.

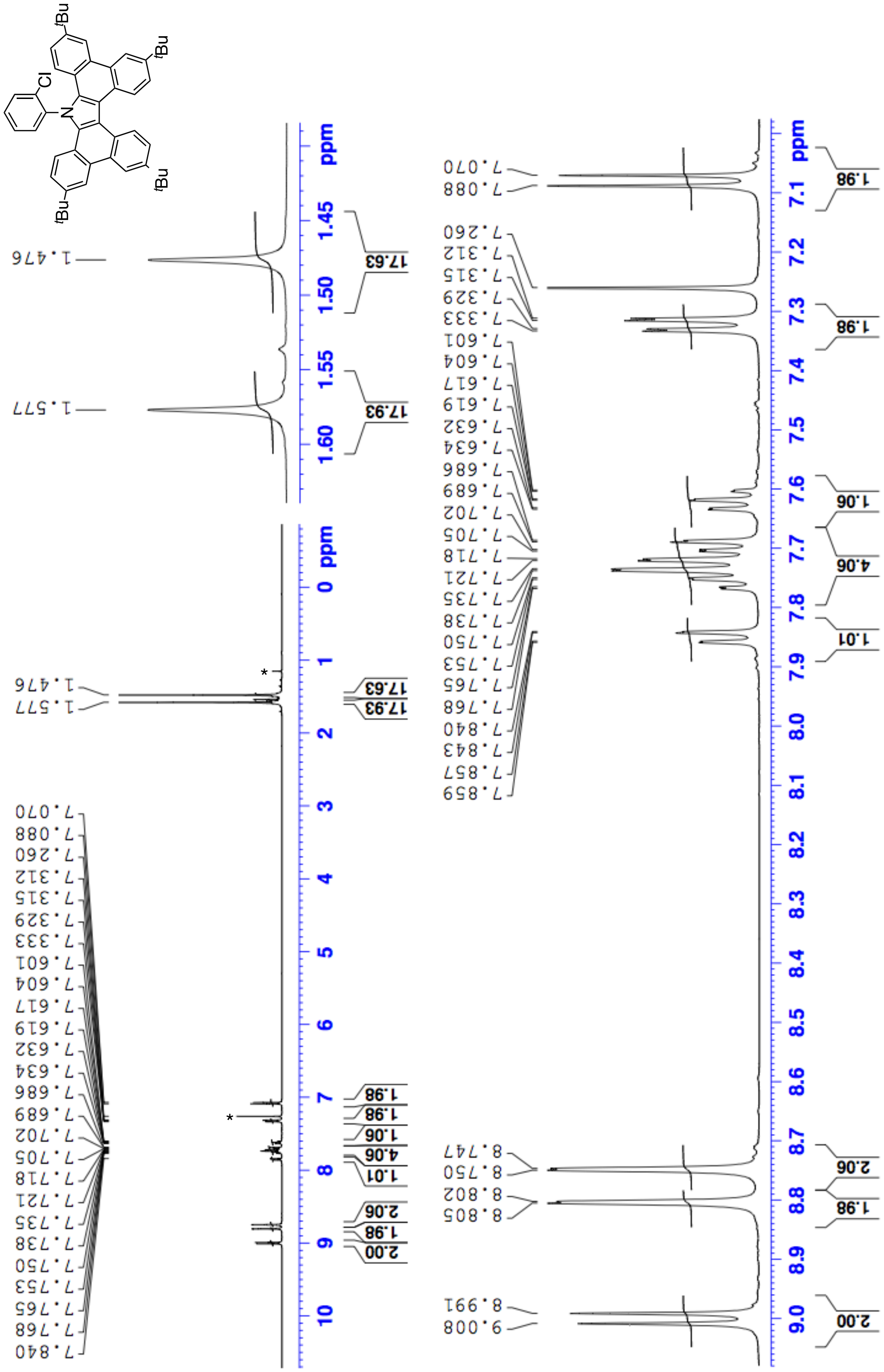

Supplementary Figure 5. ^{1}H NMR spectrum of **2** in $CDCl_3$.

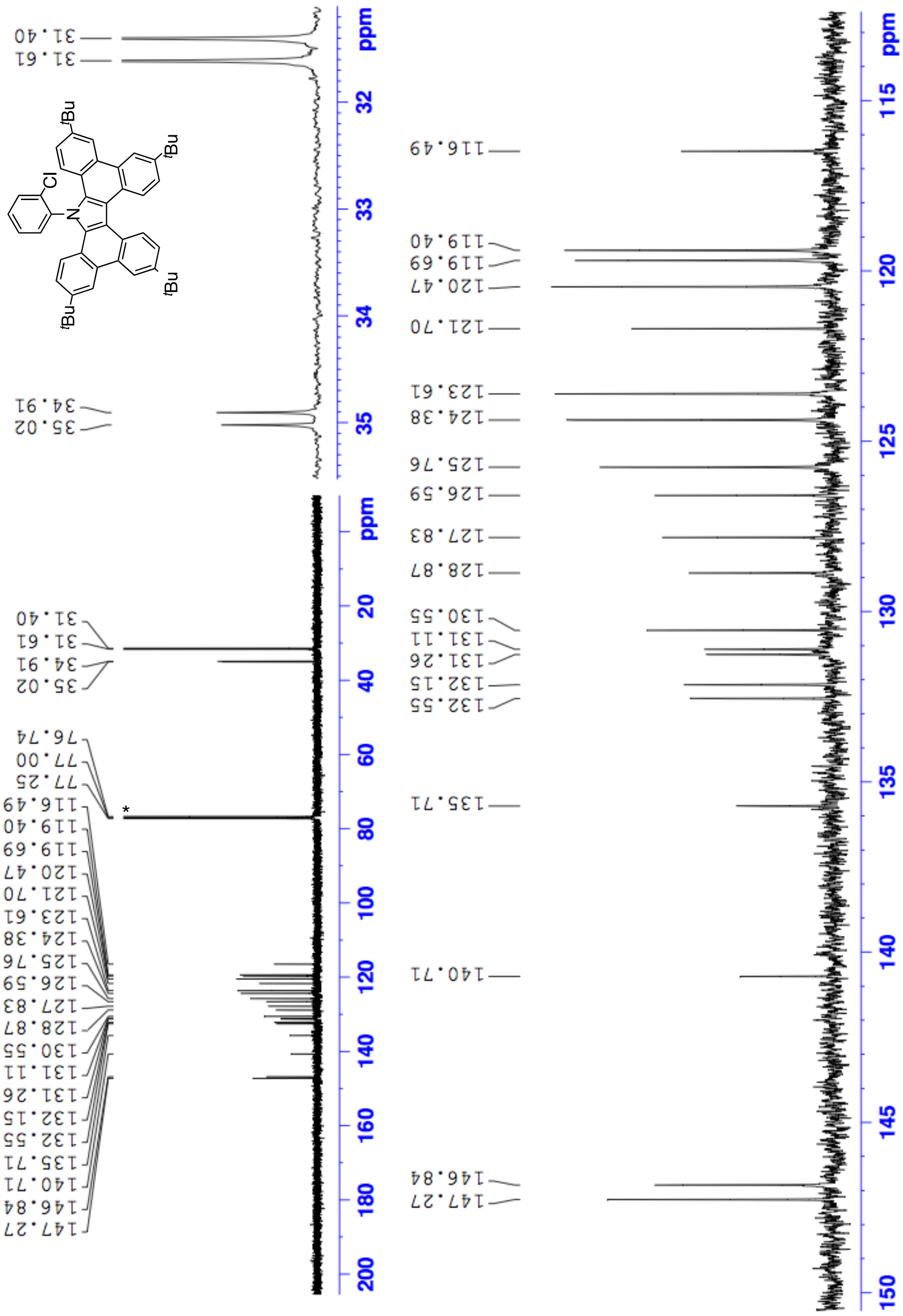

Supplementary Figure 6. ^{13}C NMR spectrum of **2** in $CDCl_3$.

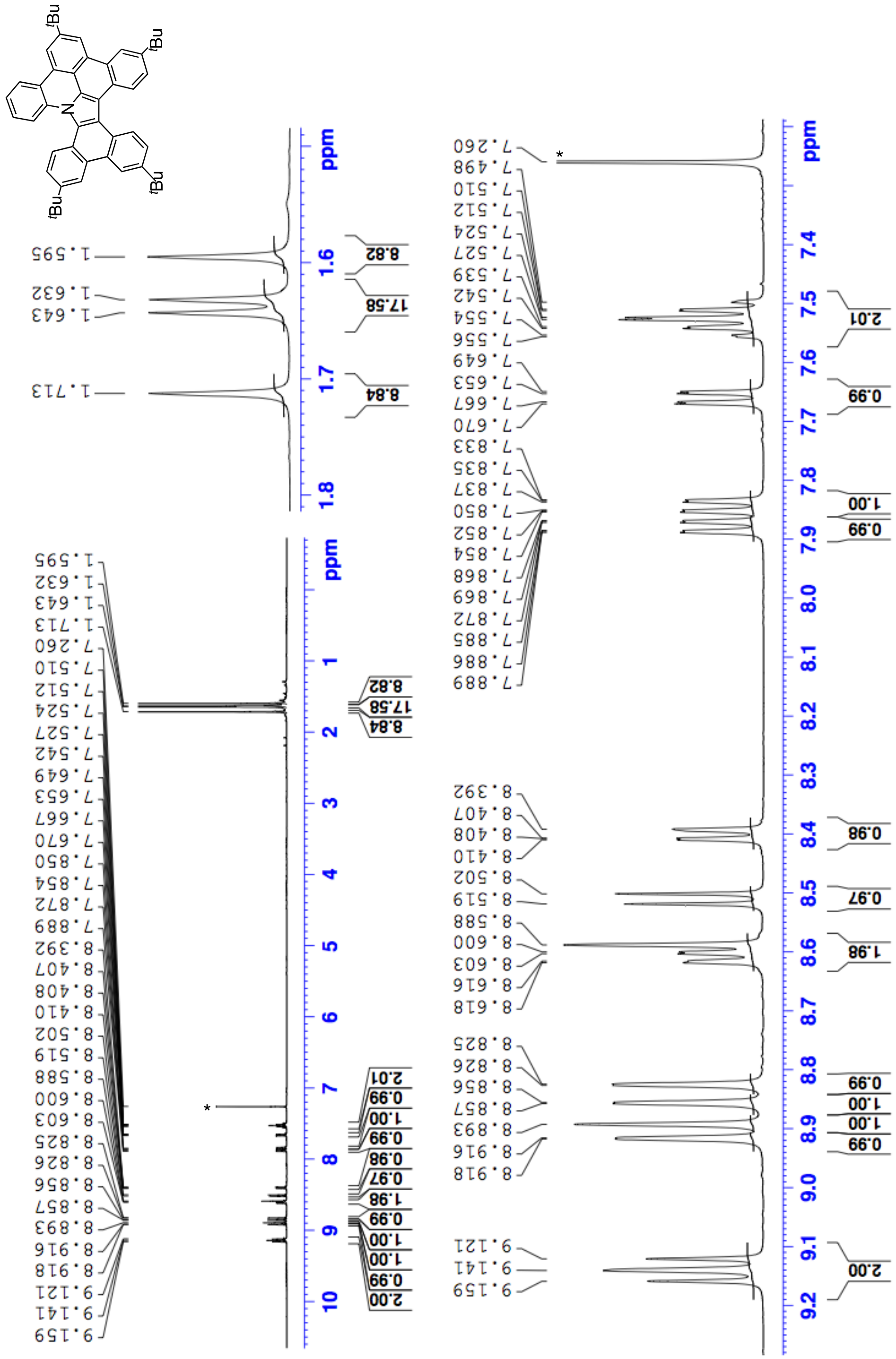

Supplementary Figure 7. ^{1}H NMR spectrum of **3** in $CDCl_3$.

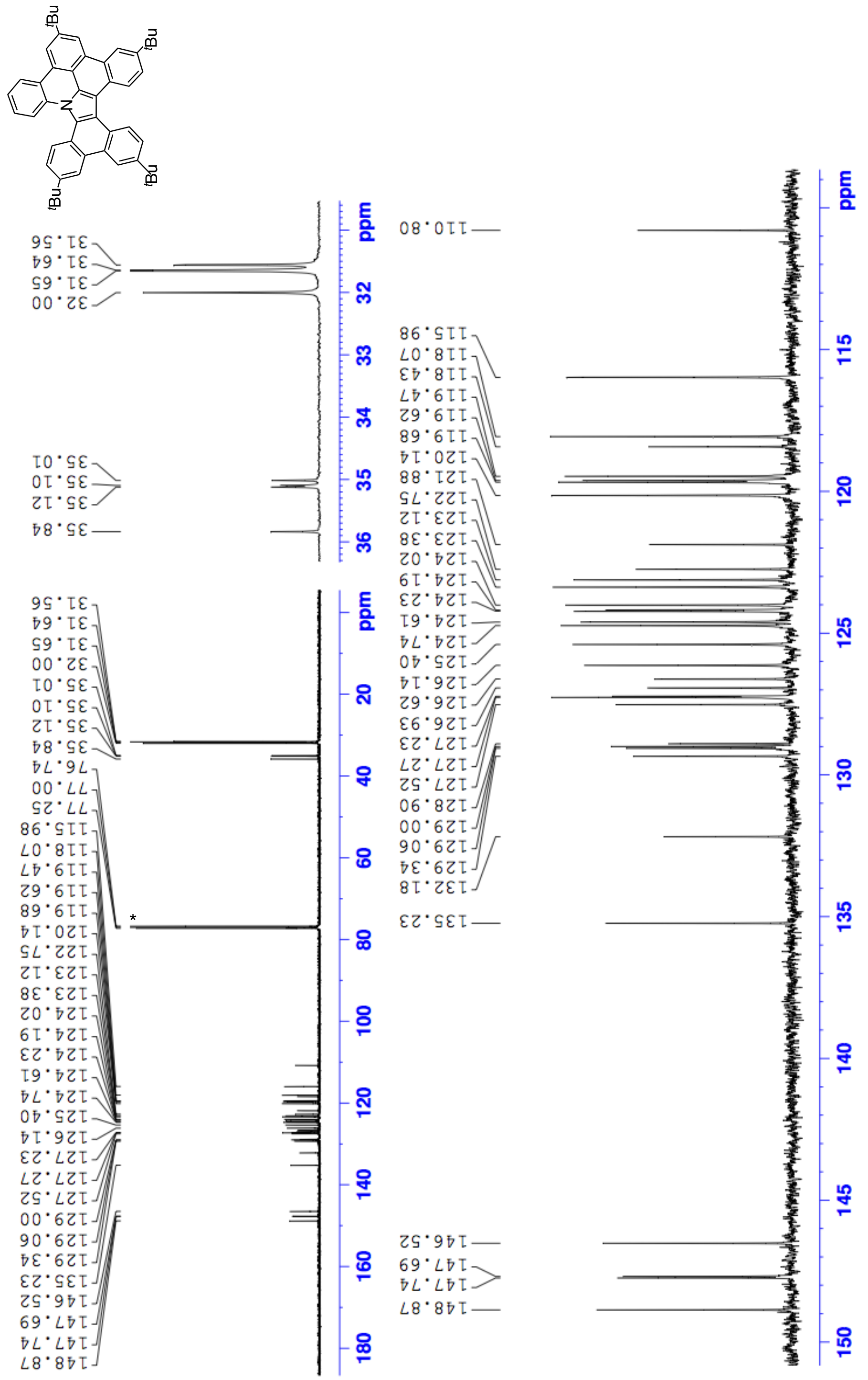

Supplementary Figure 8. ^{13}C NMR spectrum of **3** in $CDCl_3$.

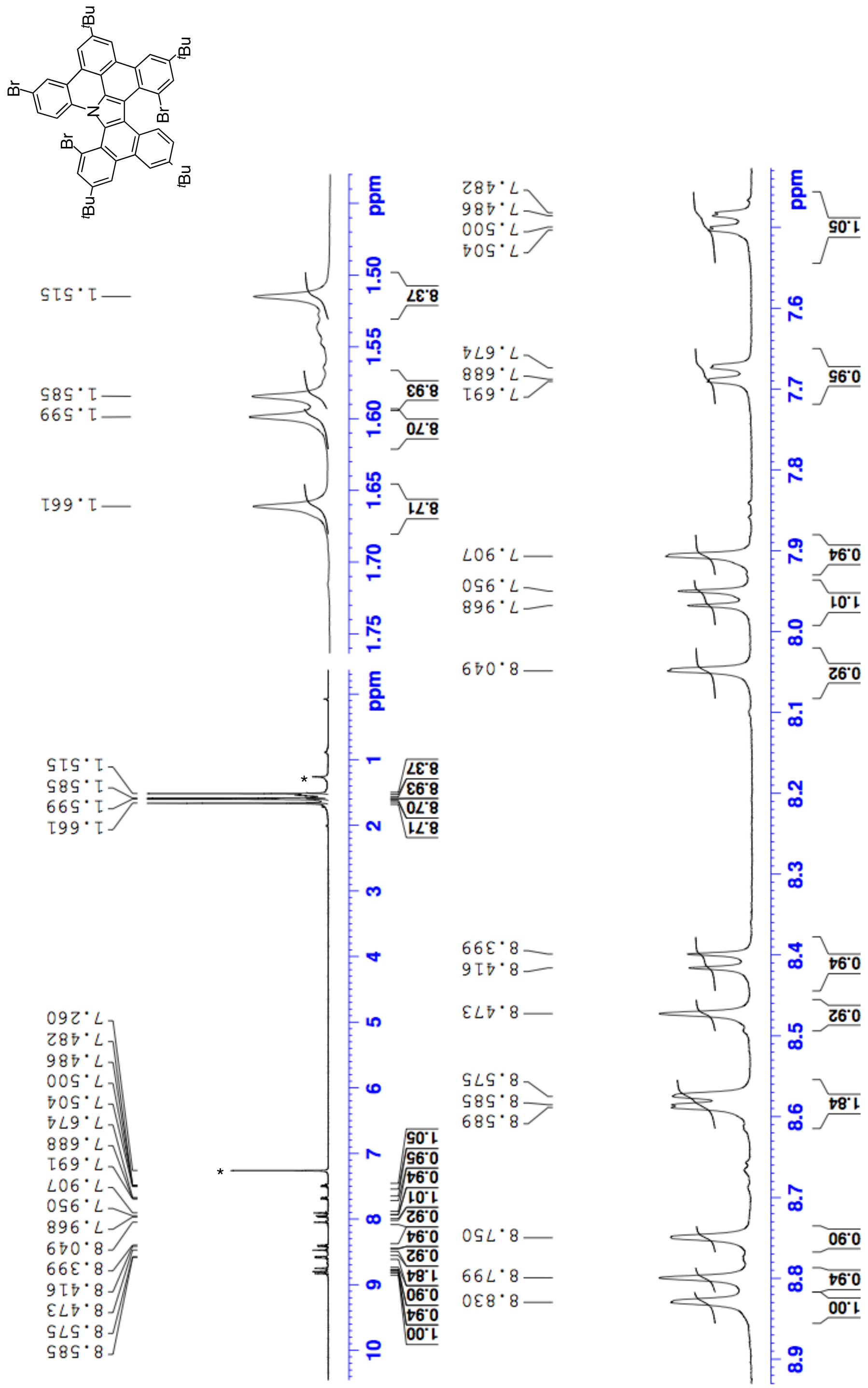

Supplementary Figure 9. ^{1}H NMR spectrum of **4** in $CDCl_3$.

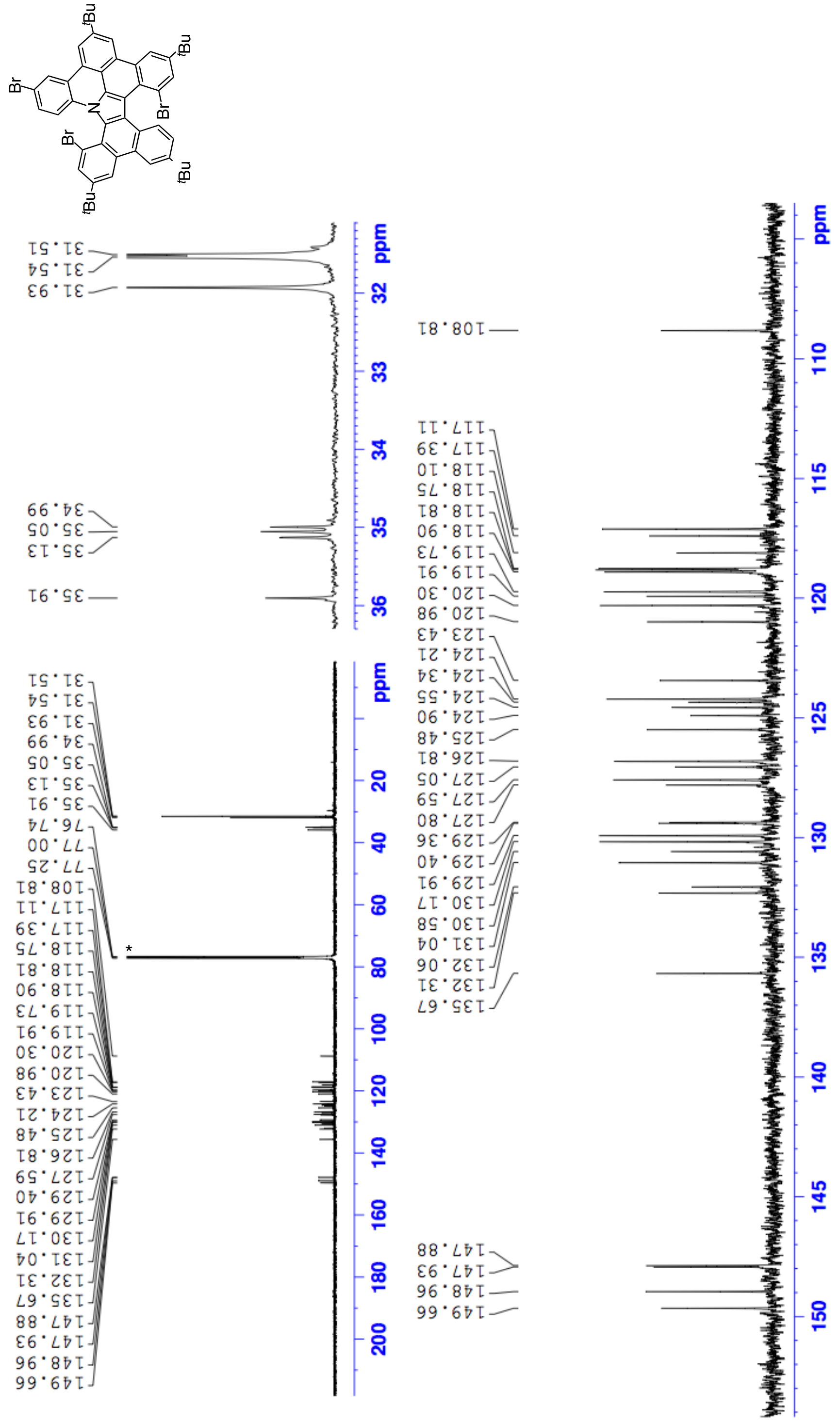

Supplementary Figure 10. ^{13}C NMR spectrum of **4** in $CDCl_3$.

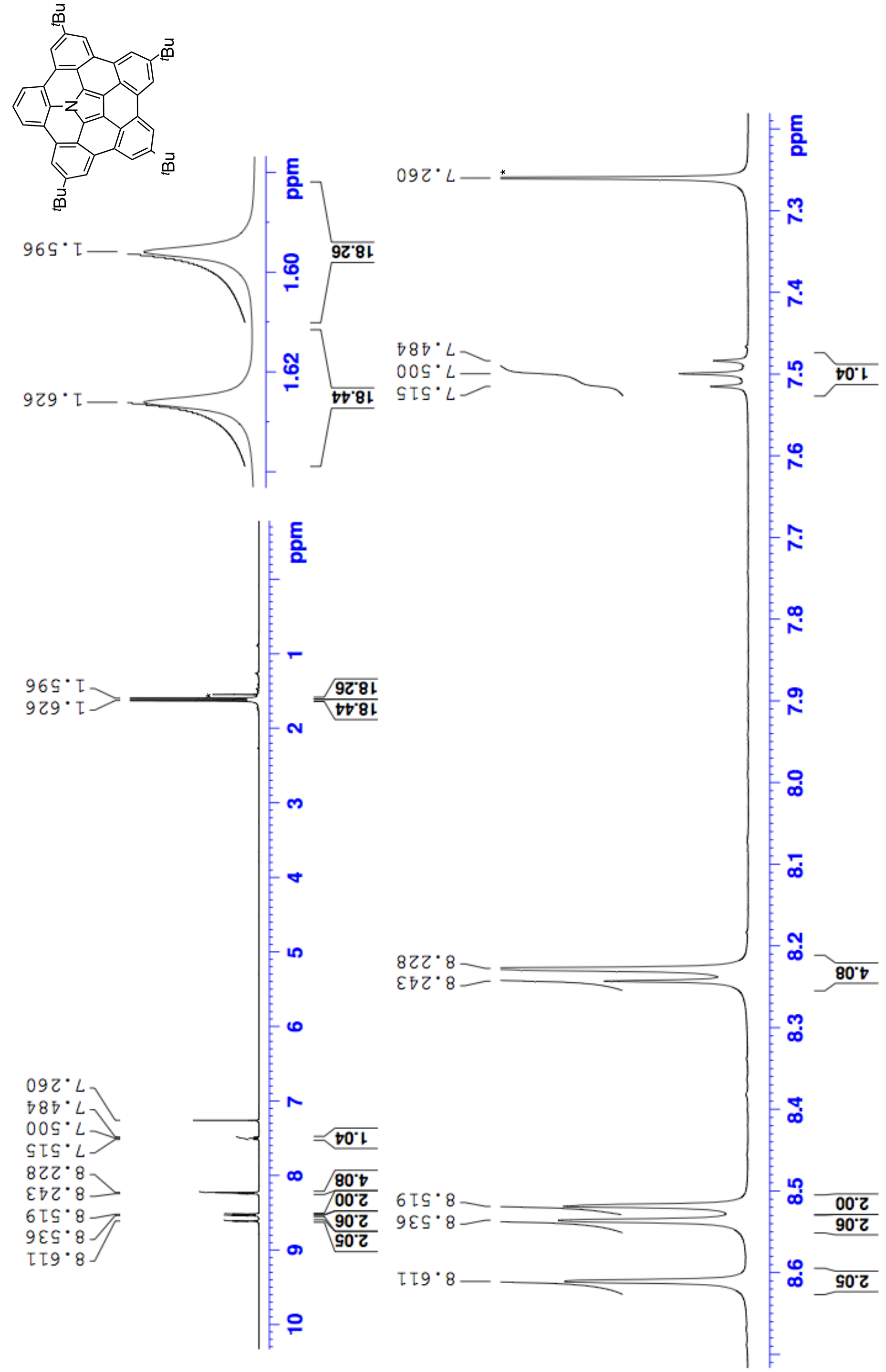

Supplementary Figure 11. ^{1}H NMR spectrum of **5** in $CDCl_3$.

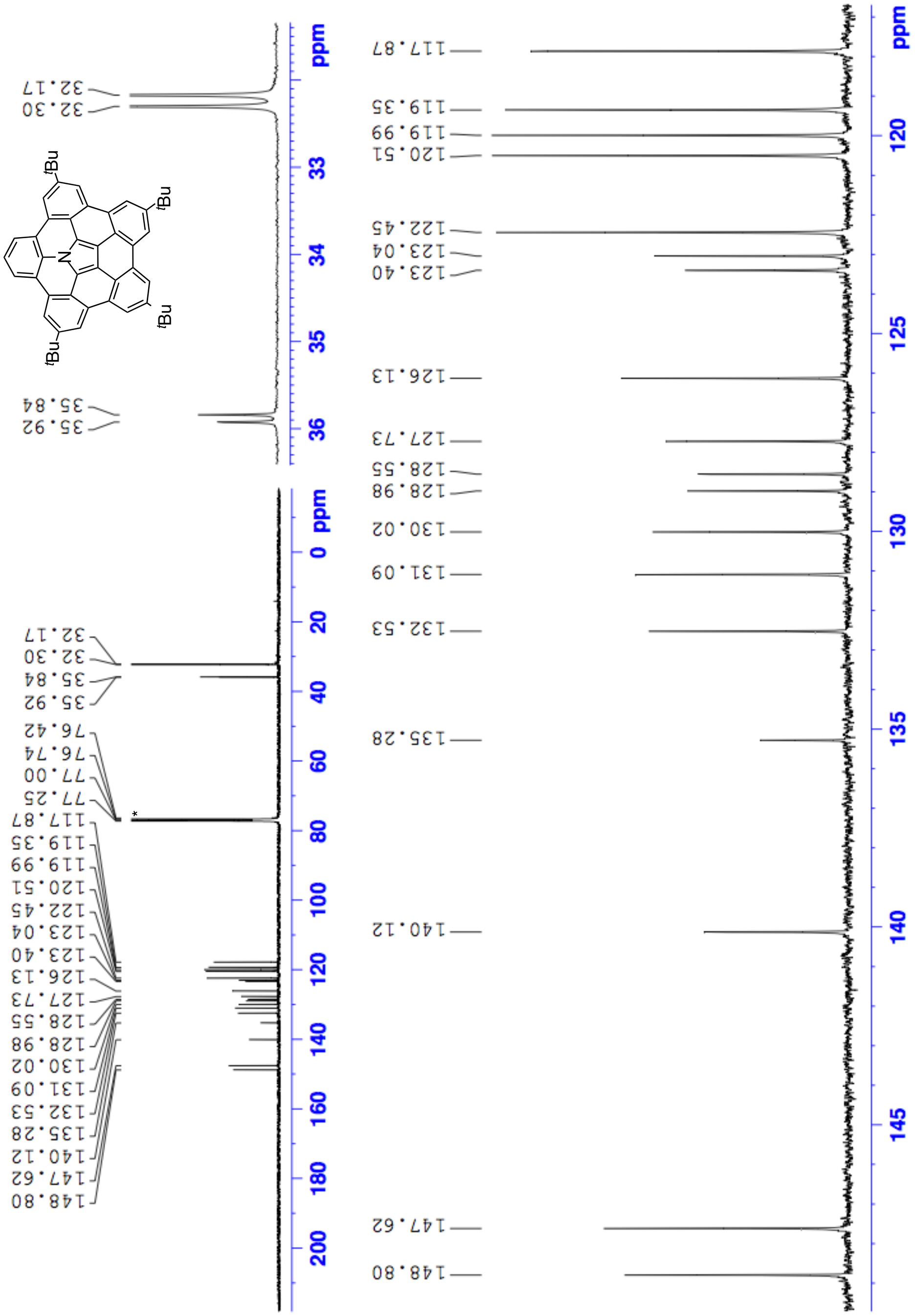

Supplementary Figure 12. ^{13}C NMR spectrum of **5** in $CDCl_3$.

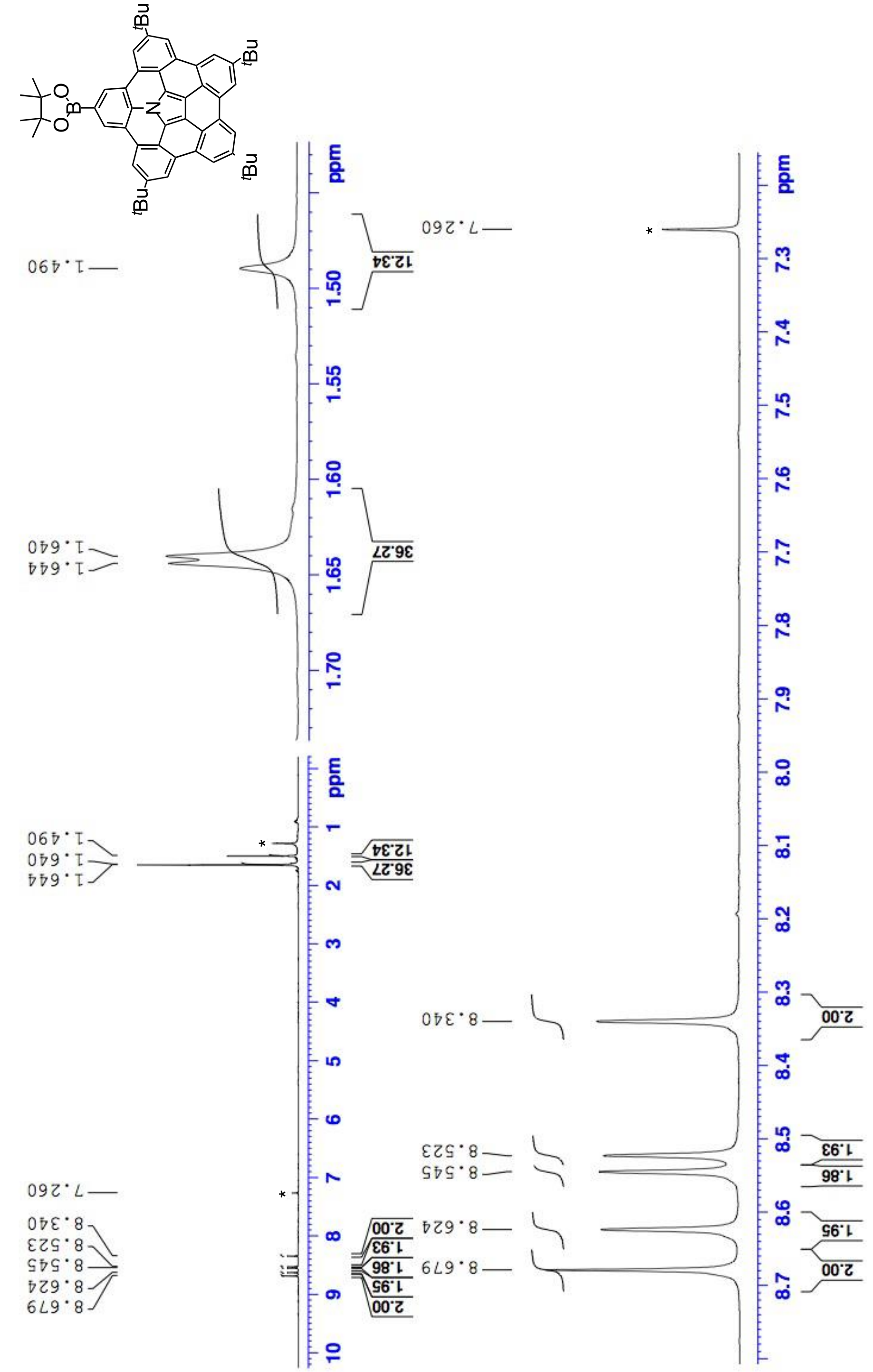

Supplementary Figure 13. ^{1}H NMR spectrum of **6** in $CDCl_3$.

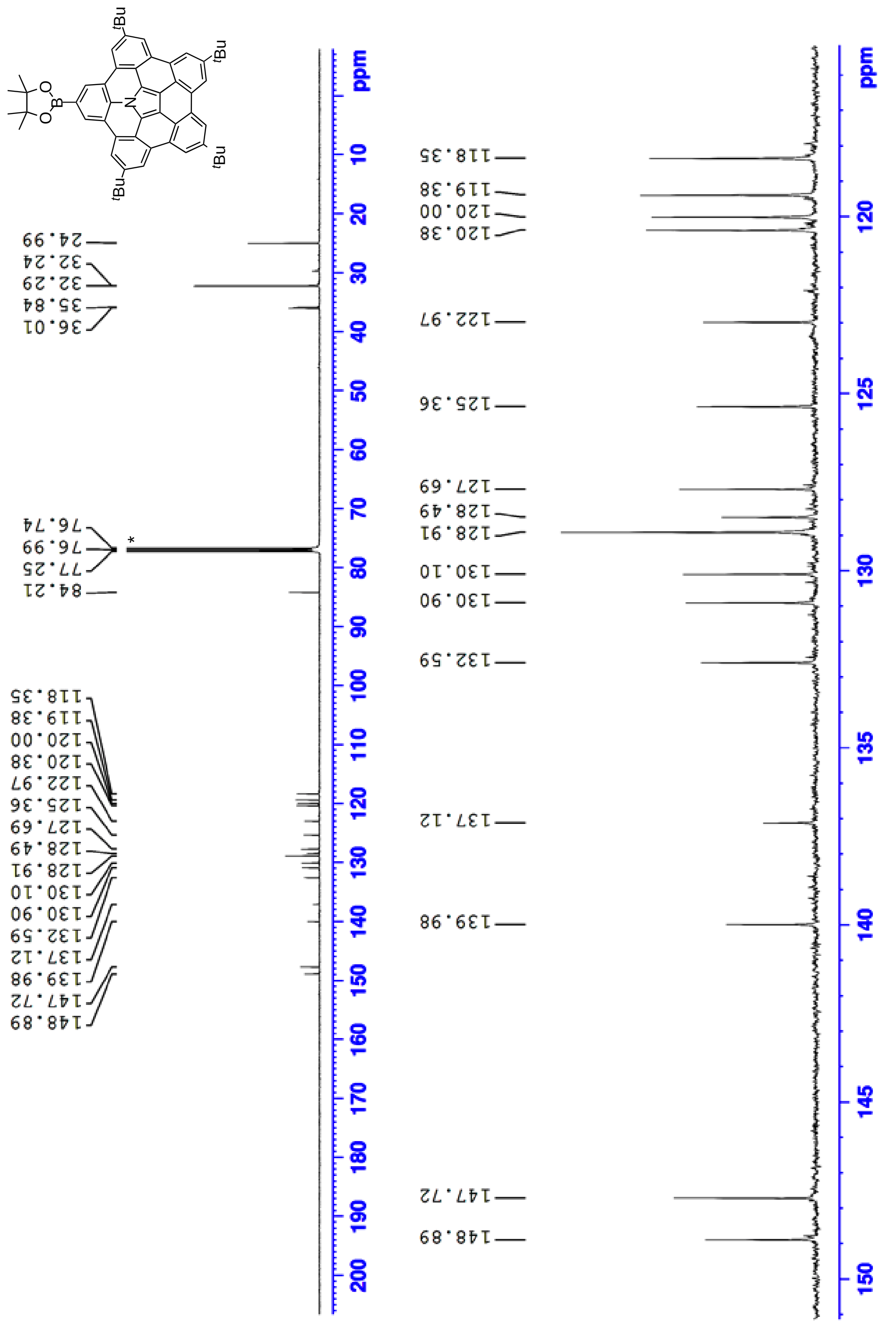

Supplementary Figure 14. ^{13}C NMR spectrum of **6** in $CDCl_3$.

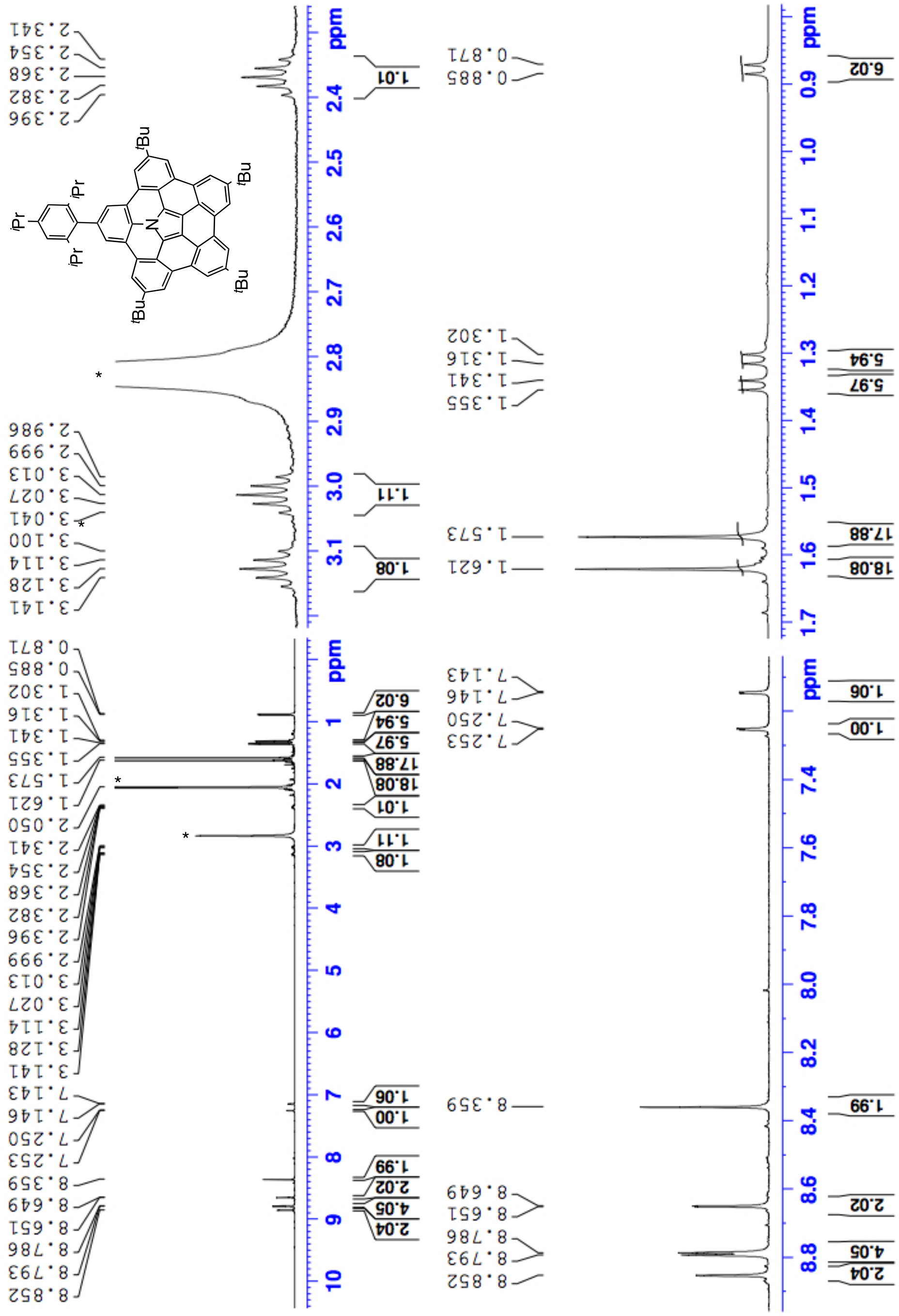

Supplementary Figure 15. ^{1}H NMR spectrum of **7** in acetone-d_6.

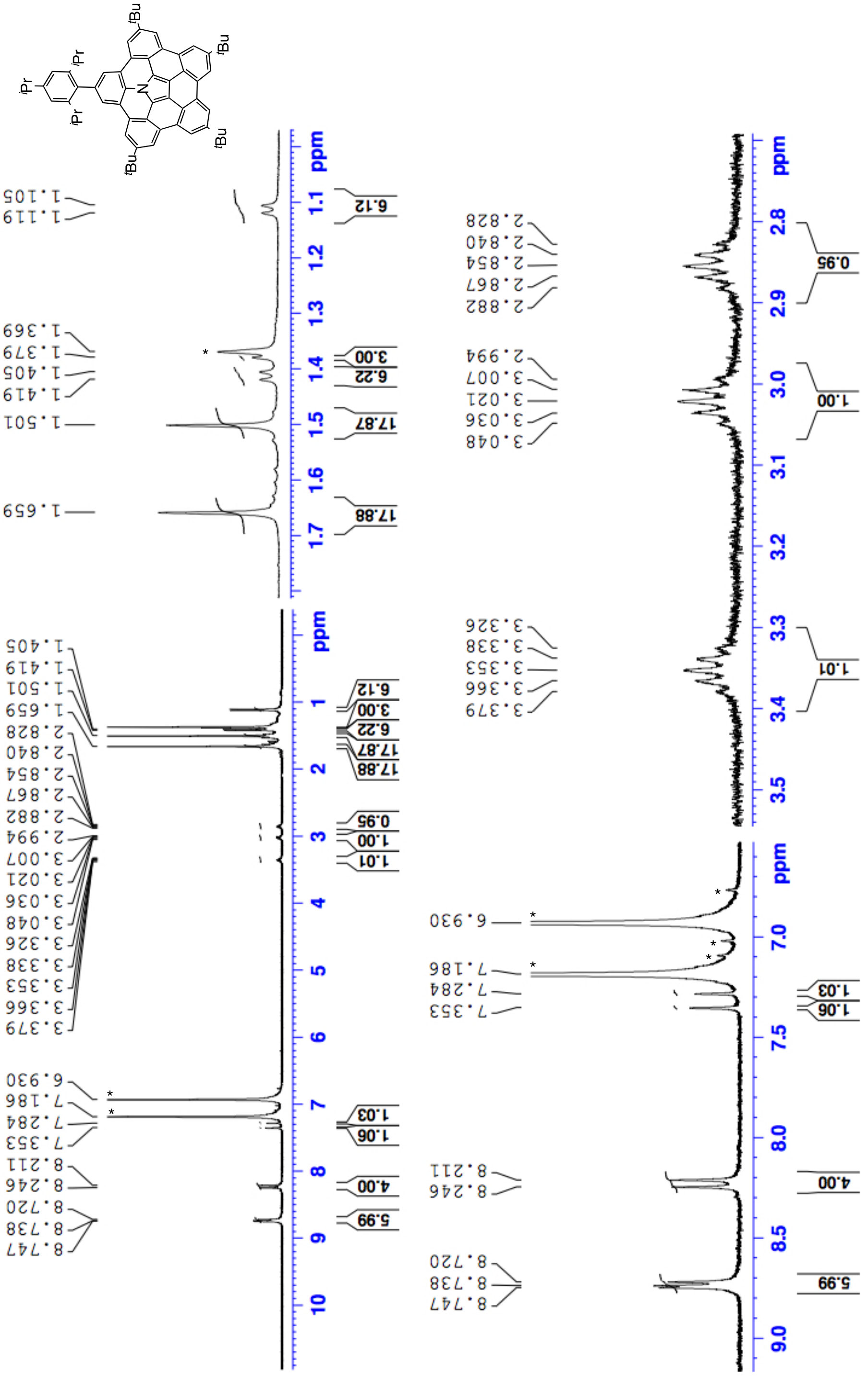

Supplementary Figure 16. ^{1}H NMR spectrum of **7** in 1,2-dichlorobenzene-d_4.

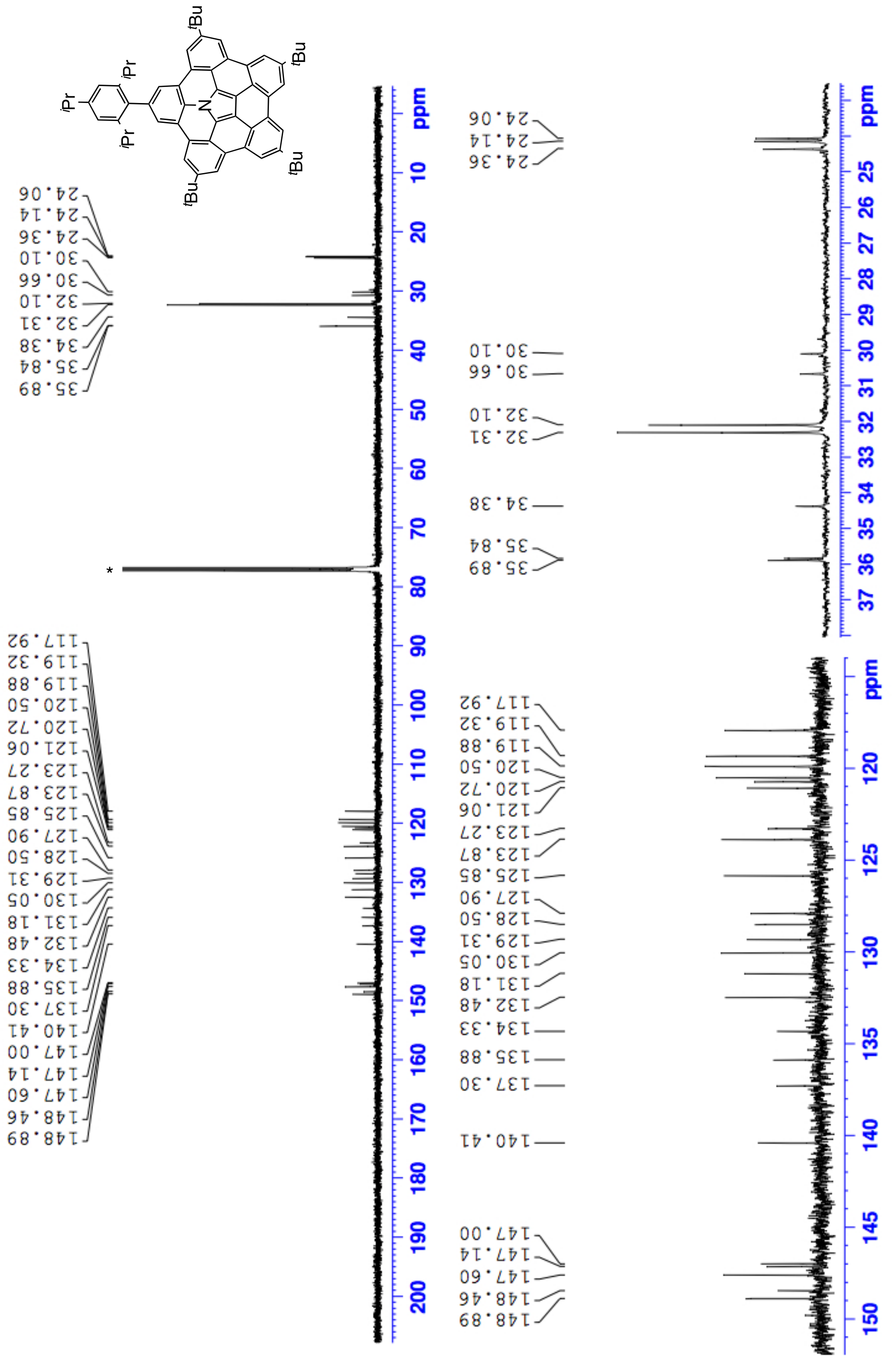

Supplementary Figure 17. ^{13}C NMR spectrum of **7** in $CDCl_3$.

a. **b.**

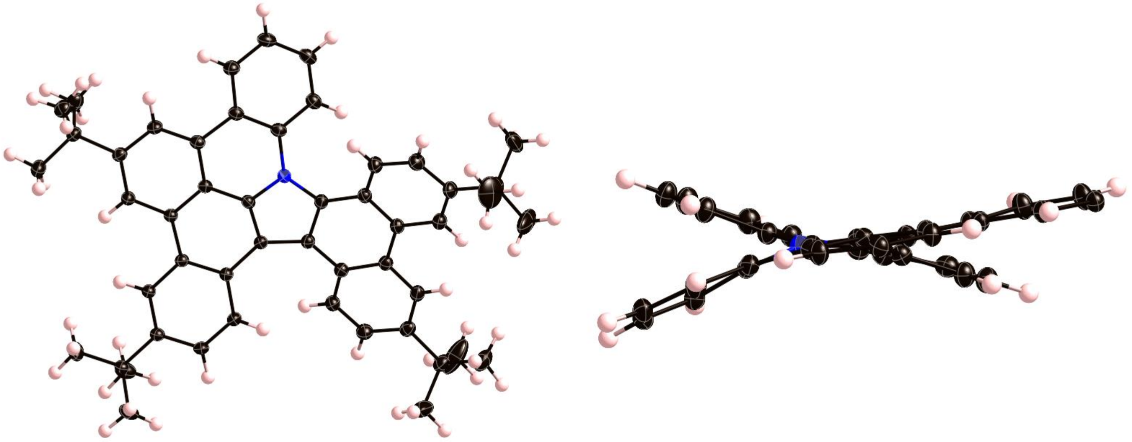

Supplementary Figure 18. X-ray crystal structure of **3**. (**a**) Top view and (**b**) side view. The thermal ellipsoids are scaled at 50% probability level. The *tert*-butyl substituents are omitted for clarity in Fig. b.

a. **b.**

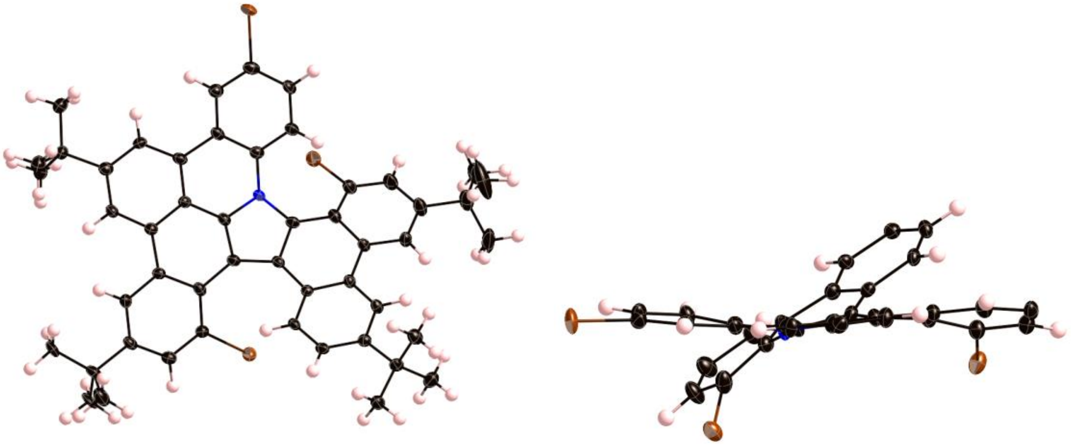

Supplementary Figure 19. X-ray crystal structure of **4**. (**a**) Top view and (**b**) side view. The thermal ellipsoids are scaled at 50% probability level. The *tert*-butyl substituents are omitted for clarity in (b).

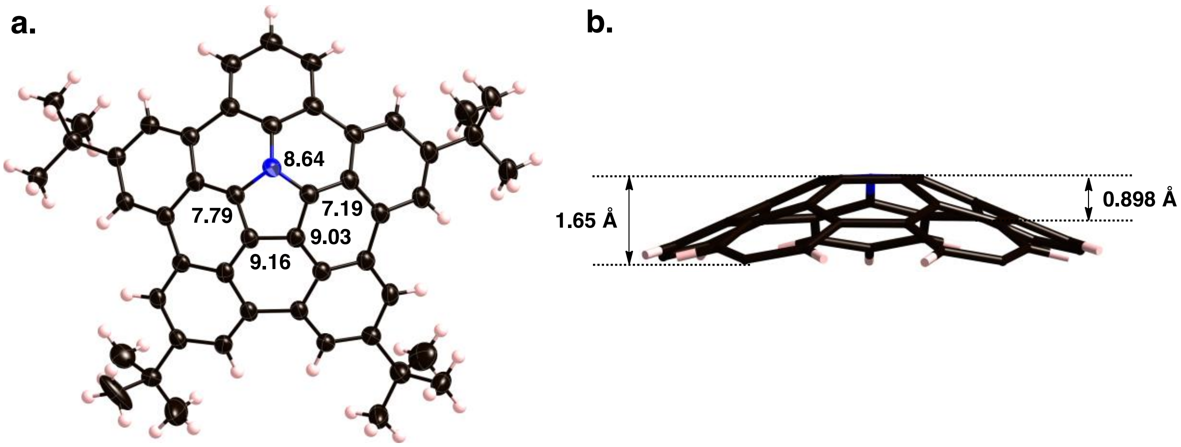

Supplementary Figure 20. X-ray crystal structure of one of two molecules of **5** in the crystal. The molecule is different from that shown in the main text (Figure 2). (**a**) Top view and (**b**) side view. The thermal ellipsoids are scaled at 50% probability level. The *tert*-butyl substituents are omitted for clarity in (b).

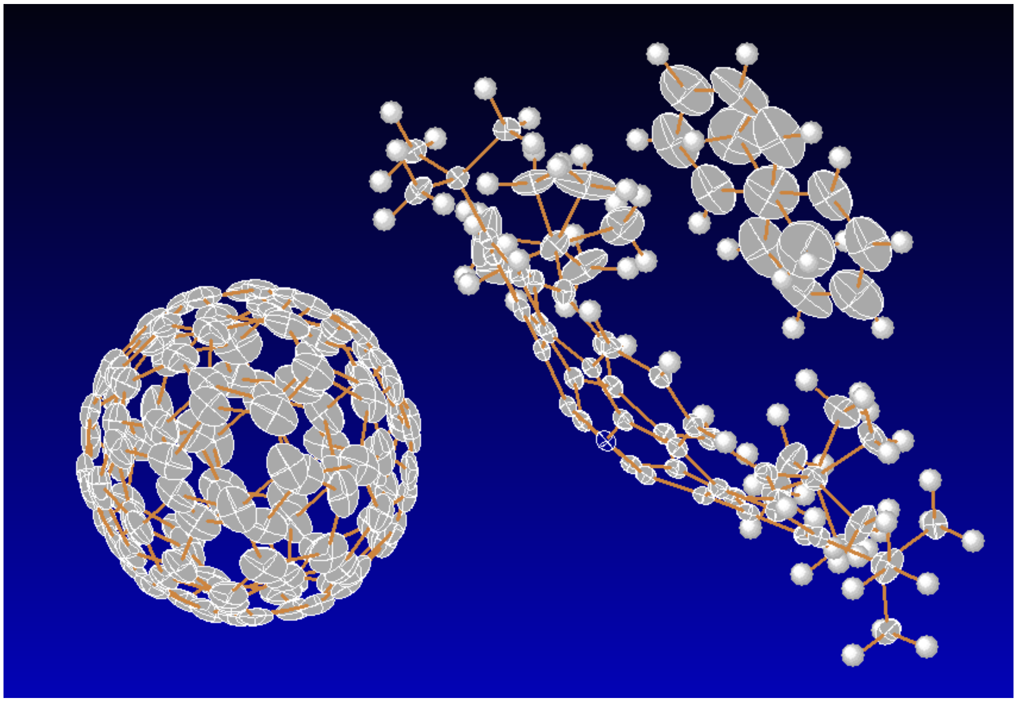

Supplementary Figure 21. X-ray crystal structure of **5⊃C$_{60}$** described in grow-up mode using XSHELL. The thermal ellipsoids were scaled at 50% probability level.

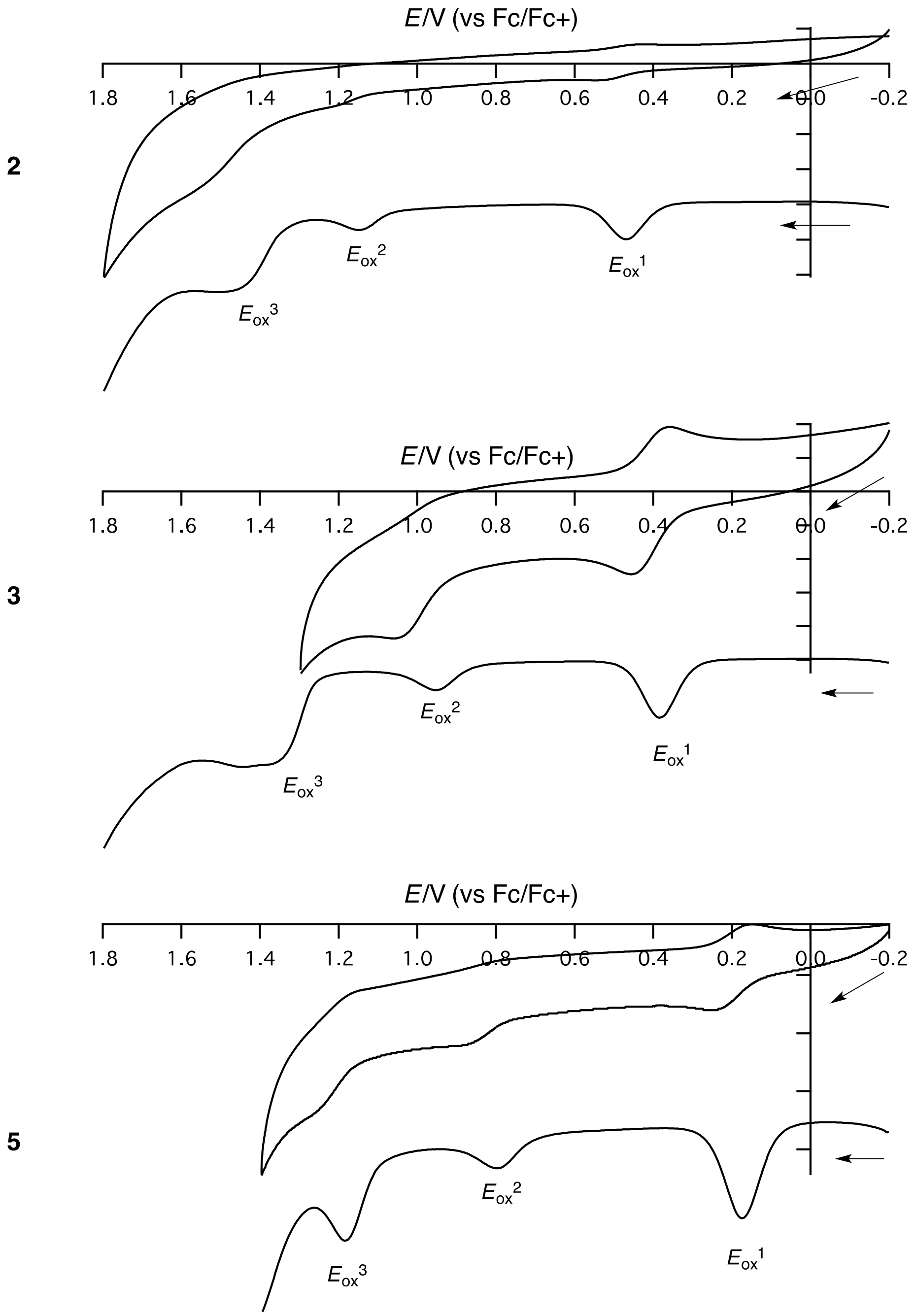

Supplementary Figure 22. Cyclic voltammograms (top) and differential pulse voltammograms (bottom) of **2**, **3** and **5**.

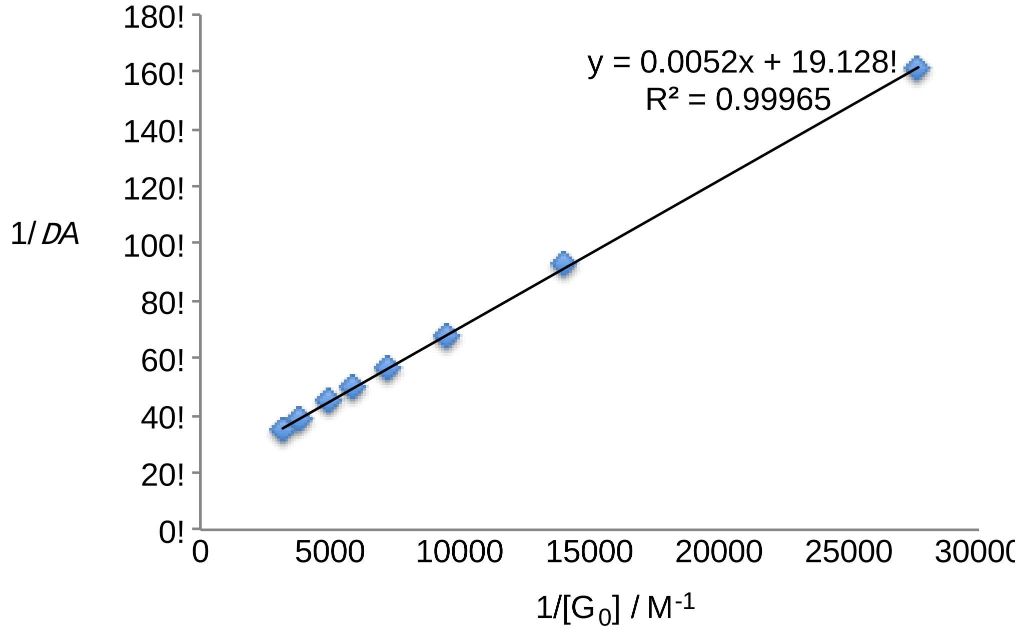

Supplementary Figure 23. Bensi-Hildebrand plot on titration of C_{60} into **5** obtained by UV/vis absorption spectral analysis.

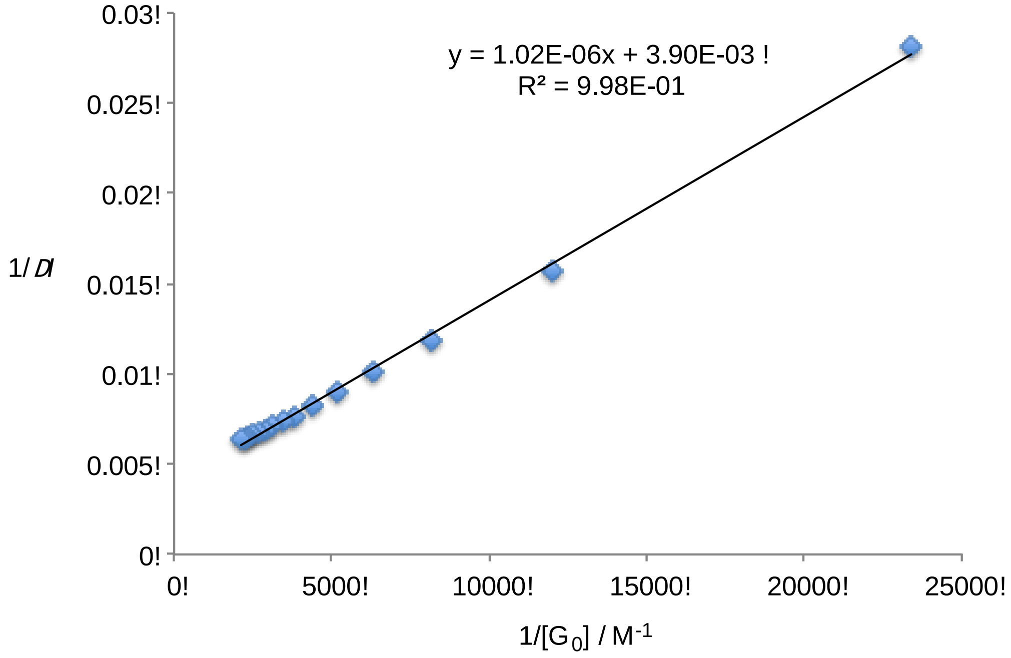

Supplementary Figure 24. Bensi-Hildebrand plot on titration of C_{60} into **5** obtained by fluorescence spectral analysis.

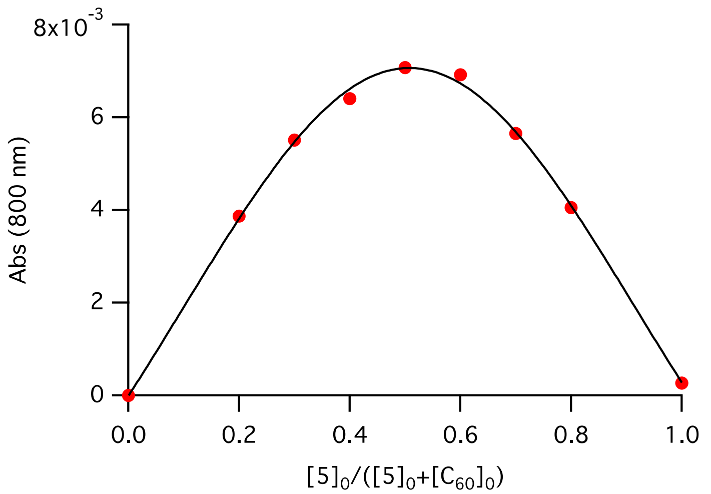

Supplementary Figure 25. Job's plot for complexation of C_{60} and **5**.

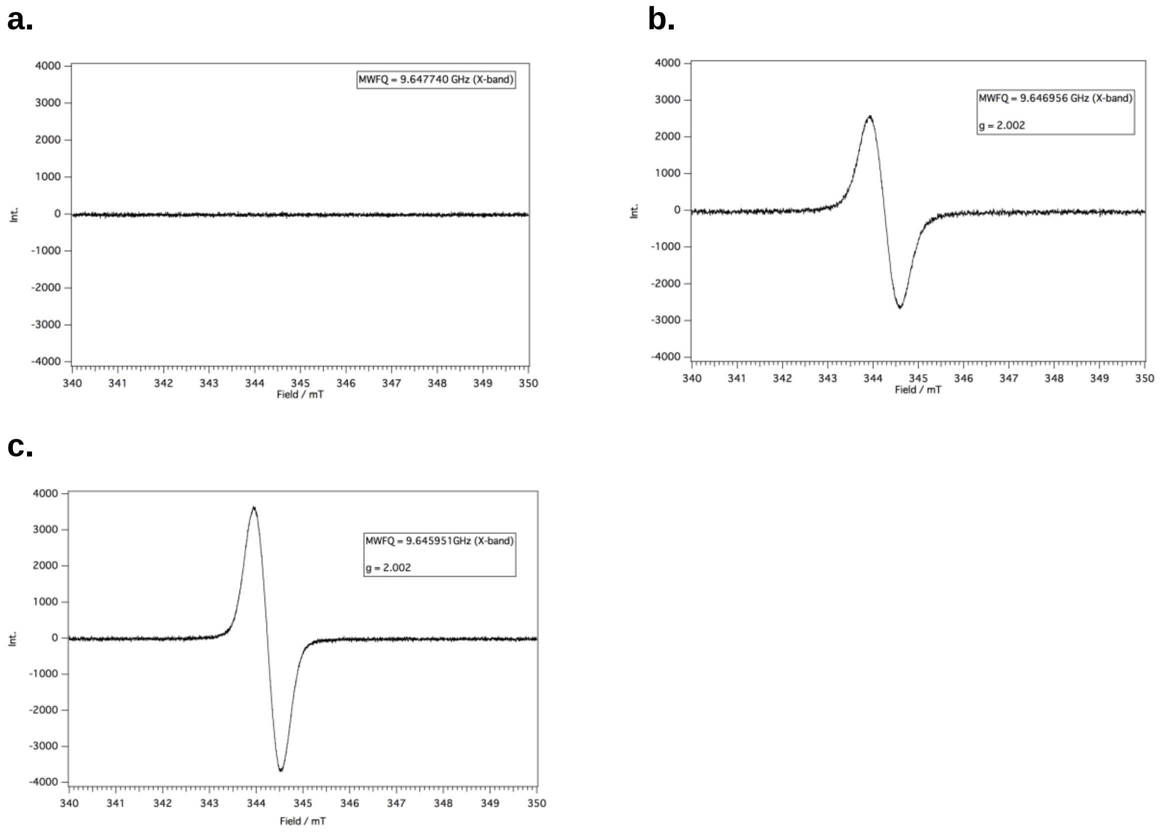

Supplementary Figure 26. ESR spectra of (**a**) compound **5** under air, (b) **5** with 10800 equiv. of TFA under air, and (c) **5** with 0.8 equiv. of BAHA under N_2 in CH_2Cl_2 solution at room temperature.

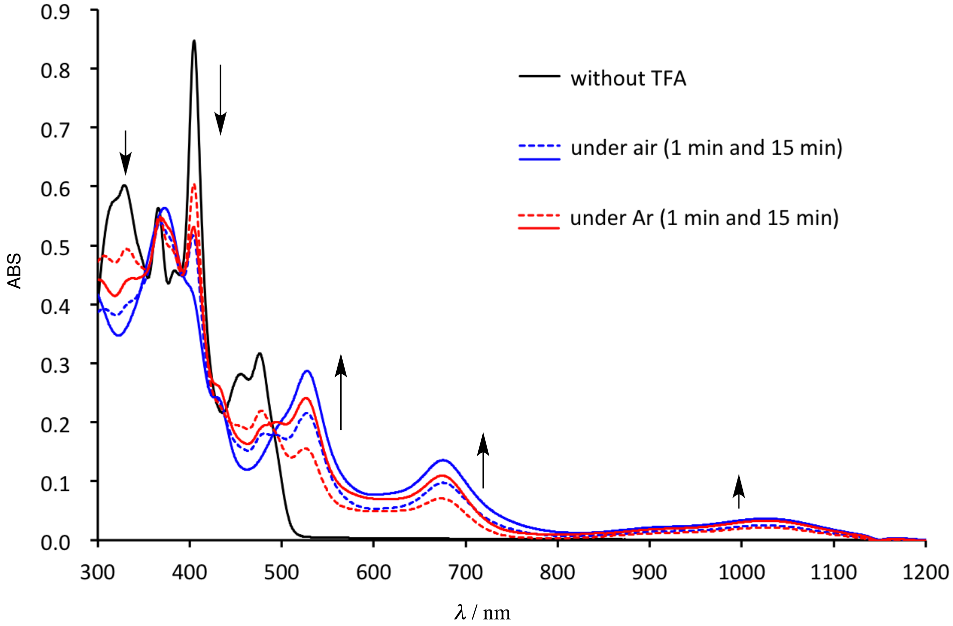

Supplementary Figure 27. UV/vis absorption spectral changes of **5** in toluene in the presence of TFA (4500 equiv.) either under air or Ar (dashed line: 1 min, solid line: 15 min).

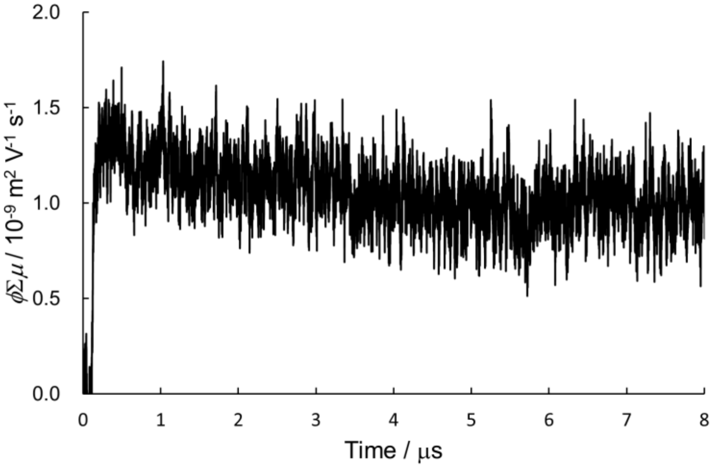

Supplementary Figure 28. Kinetic traces of a photoconductivity transient of **5** by FP-TRMC method.

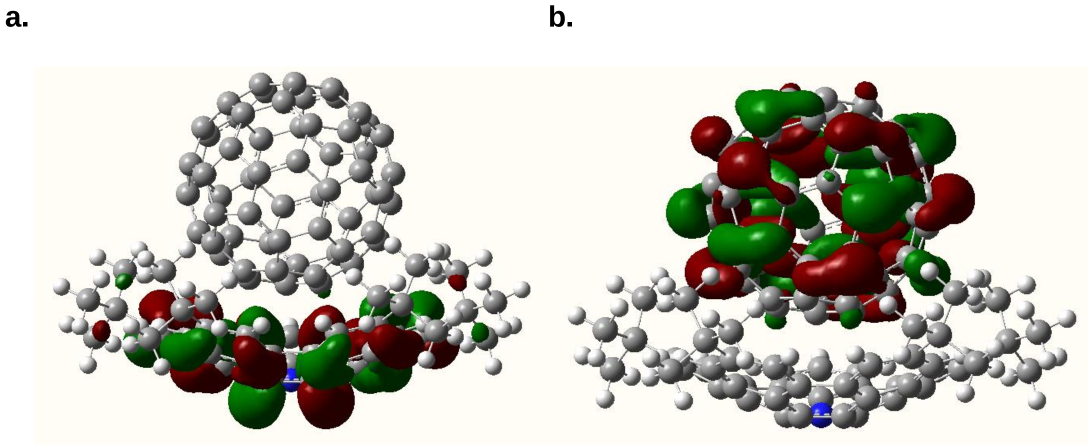

Supplementary Figure 29. (**a**) HOMO and (**b**) LUMO of **5**⊃$\mathbf{C_{60}}$.

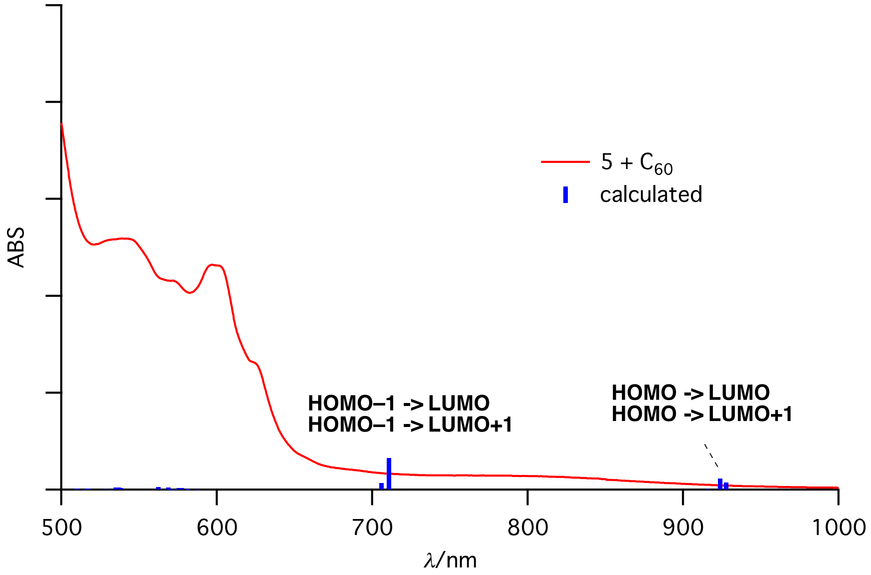

Supplementary Figure 30. Calculated oscillator strengths of **5**⊃$\mathbf{C_{60}}$ (blue stick). The spectrum of **5** in the presence of 10 equiv. of C_{60} (red line) in 1,2-dichlorobenzene was also displayed for comparison.

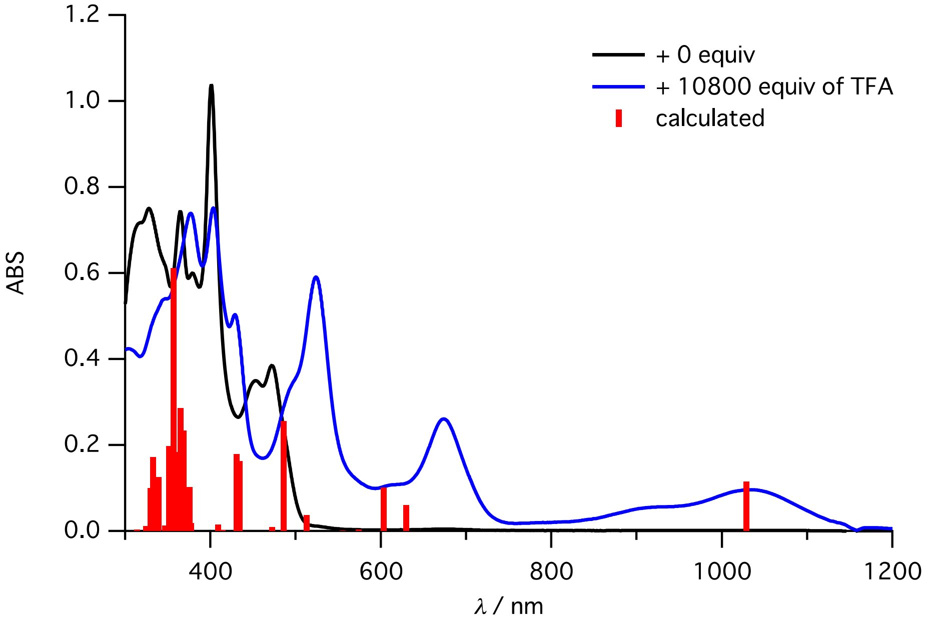

Supplementary Figure 31. Calculated oscillator strengths of radical cation $\mathbf{5}^{+\bullet}$ (red line). The spectra of **5** before (black line) and after (blue line) addition of TFA were also displayed.

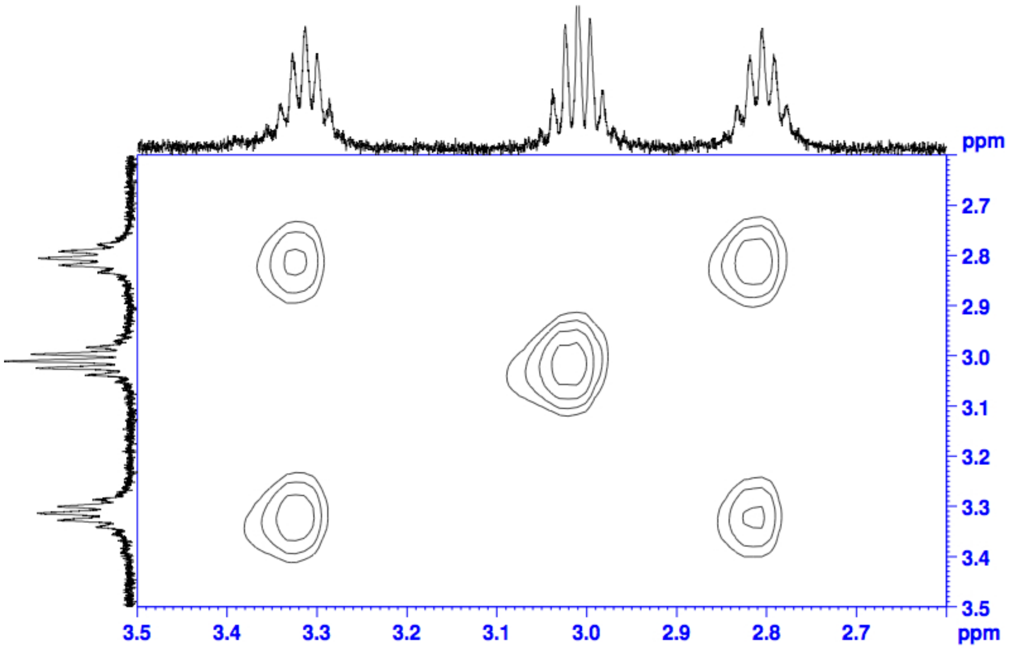

Supplementary Figure 32. 2D EXSY spectrum of **7** in 1,2-dichlorobenzene-d_4.

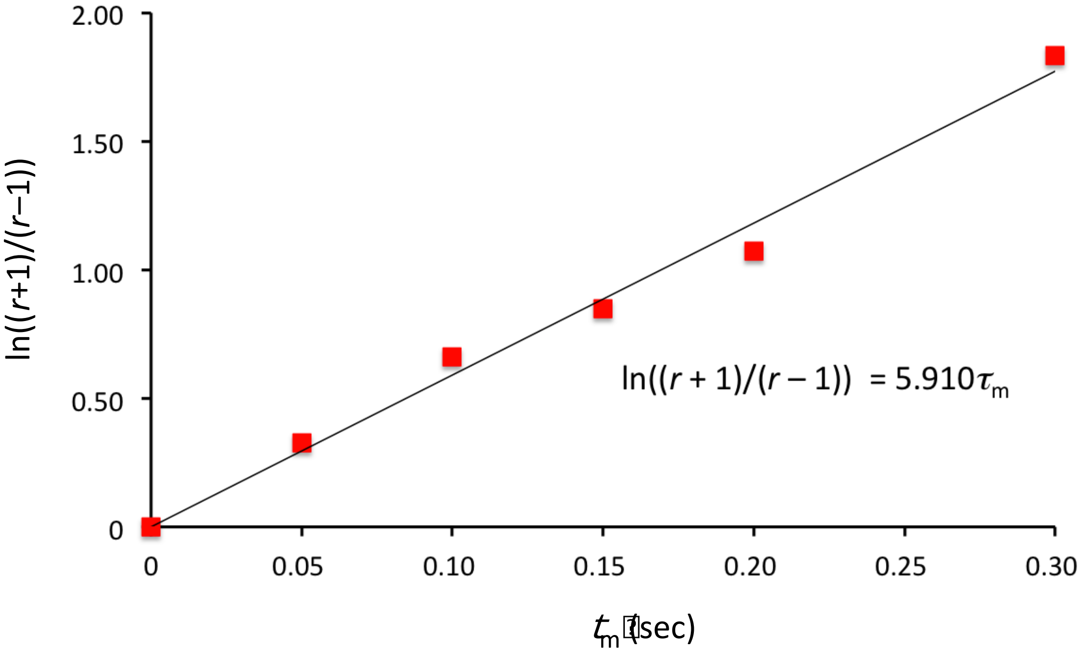

Supplementary Figure 33. Plot of 2D EXSY measurement in 1,2-dichlorobenzene-d_4.

Supplementary Table 1. Summary of Crystallographic data for **3**, **4**, **5** and **5⊃C$_{60}$**.

	**3**	**4**	**5**	**5 C$_{60}$**
empirical formula	$C_{50}H_{51}N$	$C_{55}H_{58}Br_3Cl_5N$	$C_{116}H_{114}N_2$	$C_{113.5}H_{51}N$
formula weight	665.91	1150.00	1536.09	1428.55
habit	prism	prism	prism	prism
T, K	123(2)	93(2)	103(2)	103(2)
crystal system	triclinic	triclinic	triclinic	triclinic
space group	*P*-1 (2)	*P*-1 (2)	*P*-1 (2)	*P*-1 (2)
a, Å	9.9215(5)	11.6888(11)	14.698(2)	13.340(4)
b, Å	11.5220(5)	15.2279(14)	17.333(2)	14.585(4)
c, Å	17.8122(9)	16.1361(16)	18.574(3)	17.980(5)
α, deg	104.4750(10)	94.960(3)	70.845(5)	98.107(3)
β, deg	93.8730(10)	111.005(3)	87.159(6)	101.423(6)
γ, deg	108.0950(10)	103.447(2)	80.952(6)	97.323(4)
V, Å^3	1850.60(16)	2561.8(4)	4414.3(11)	3350.0(15)
Z	2	2	2	2
Dc, g/cm^3	1.195	1.491	1.156	1.416
F(000)	716	1170	1648	1478
crystal size, mm^3	0.26 x 0.26 x 0.15	0.46 x 0.34 x 0.19	0.30 x 0.14 x 0.08	0.44 x 0.28 x 0.12
$2\theta_{max}$, °	50.0	50.0	50.0	50.0
R_1 ($I > 2\sigma(I)$)	0.0433	0.0600	0.0728	0.0561
wR_2 (all data)	0.1231	0.1553	0.2161	0.1682
GOF	1.017	1.087	1.064	1.065
obs reflects	6295	8382	15464	11448
total reflects	12793	14718	43659	22169
parameters	472	669	1195	1645

Supplementary Table 2. Oxidation potentials of **2**, **3** and **5**.

Compound	E_{ox}^3	E_{ox}^2	E_{ox}^1
**2**	1.50	1.15	0..489
**3**	—	0.954	0.408
**5**	1.19	0.80	0.20

Supplementary Table 3. Atom list for optimized structure of **5⊃C_{60}** at the M06-2X/6-31G(d) level.

Symbol	X	Y	Z
H	2.242237	-1.310563	-5.54244
H	0.11643	-0.76163	-6.633029
H	-2.033261	-1.342908	-5.60883
H	3.940209	-1.42618	-4.203351
H	5.228287	-2.376026	-0.233691
H	4.515098	-2.747576	1.743292
H	1.011063	-3.346075	4.118433
H	-1.08914	-3.44061	4.098038
H	-4.527939	-2.893973	1.616079
H	-5.134554	-2.39303	-0.365834
H	-3.756231	-1.449874	-4.306433
H	7.015472	-2.895903	-3.52313
H	7.700901	-1.32531	-3.991254
H	6.147692	-1.851526	-4.658897
H	6.573363	-0.662296	-0.514467
H	7.931656	-0.643433	-1.646342
H	7.291012	-2.190773	-1.075357
H	5.101145	0.358681	-3.818257
H	6.662141	0.830755	-3.134103
H	5.218319	0.774995	-2.101647
H	4.814778	-1.159677	3.220858
H	4.844005	-1.008341	4.984766
H	3.384519	-0.564697	4.078791
H	4.534553	-4.708676	4.564159
H	5.479744	-3.408772	5.321465
H	5.538879	-3.645451	3.56742
H	2.031765	-2.063464	5.559115

H	3.506365	-2.491901	6.434743
H	2.47695	-3.772278	5.776269
H	-2.219921	-2.221788	5.526827
H	-2.562248	-3.96011	5.691876
H	-3.688501	-2.764406	6.349502
H	-4.500964	-5.000936	4.388072
H	-5.546886	-3.984686	3.384602
H	-5.563412	-3.795374	5.146279
H	-5.075586	-1.337956	4.916421
H	-5.01783	-1.444572	3.150702
H	-3.638505	-0.779	4.03572
H	-5.133978	0.78614	-2.264415
H	-6.529597	0.782489	-3.360344
H	-4.930022	0.33029	-3.963599
H	-5.969689	-1.897976	-4.80229
H	-7.537555	-1.382119	-4.160276
H	-6.839109	-2.938539	-3.664147
H	-7.173906	-2.191523	-1.23676
H	-7.799089	-0.646981	-1.831297
H	-6.457523	-0.660857	-0.679079
C	0.084353	-2.922138	-3.498482
C	1.347039	-2.461437	-3.95354
C	1.317078	-1.688512	-5.118915
C	0.107103	-1.358324	-5.726831
C	-1.1162	-1.706579	-5.156151
C	-1.170606	-2.477953	-3.991128
C	2.40069	-3.293089	-1.841689
C	2.554137	-2.621085	-3.082328
C	3.764087	-1.967202	-3.278333
C	4.754936	-1.932516	-2.276165

C	4.496342	-2.496843	-1.023807
C	3.291656	-3.159356	-0.739699
C	1.485813	-3.955934	0.825358
C	2.815138	-3.47893	0.640973
C	3.492976	-3.110232	1.80866
C	2.870689	-3.128442	3.06429
C	1.506083	-3.451901	3.159946
C	0.760205	-3.846414	2.04628
C	-1.432334	-3.982794	0.779341
C	-0.748584	-3.872241	2.023293
C	-1.545049	-3.533009	3.119201
C	-2.916748	-3.25586	2.983909
C	-3.494438	-3.216661	1.707595
C	-2.764839	-3.536442	0.556085
C	-2.27683	-3.326256	-1.91283
C	-3.198376	-3.200794	-0.834973
C	-4.388284	-2.520458	-1.141296
C	-4.615107	-1.949202	-2.397223
C	-3.603964	-1.993765	-3.378888
C	-2.400327	-2.650135	-3.154882
C	6.057761	-1.181364	-2.574039
C	6.7703	-1.855147	-3.758769
C	7.013558	-1.175112	-1.376903
C	5.736402	0.280778	-2.930308
C	3.691641	-2.715394	4.294791
C	4.214861	-1.27787	4.12884
C	4.882662	-3.677672	4.442774
C	2.870592	-2.767804	5.587369
C	-3.799929	-2.943982	4.200486
C	-3.013027	-2.977896	5.514883

C	-4.92012	-3.995054	4.28181
C	-4.420526	-1.544424	4.062354
C	-5.913348	-1.200471	-2.725457
C	-5.602045	0.258956	-3.102745
C	-6.603686	-1.897927	-3.910101
C	-6.88734	-1.179904	-1.543159
C	-1.048942	-3.92582	-1.620501
C	-0.651916	-4.265139	-0.350024
C	0.74714	-4.260559	-0.327803
C	1.174497	-3.911753	-1.586111
C	0.819108	-0.86395	0.859359
C	-0.521661	-0.800385	1.408379
C	-1.607171	-0.631213	0.565099
C	-1.401067	-0.516433	-0.865797
C	-0.119525	-0.576659	-1.39124
C	1.017085	-0.755376	-0.508243
C	1.712882	-0.186788	1.778019
C	0.921597	0.296329	2.896867
C	-0.460767	-0.08625	2.668422
C	-1.489436	0.775077	3.024698
C	-2.687538	0.261594	0.936802
C	-2.351656	0.449037	-1.381833
C	-1.975628	1.315242	-2.39902
C	-0.632382	1.2527	-2.948254
C	0.275891	0.327433	-2.454655
C	1.658769	0.708915	-2.227347
C	2.115423	0.040125	-1.022727
C	2.970947	0.689559	-0.144228
C	2.764761	0.574204	1.288503
C	1.213531	1.525056	3.473

C	2.31134	2.324681	2.958705
C	3.067176	1.860188	1.891264
C	3.460649	2.770291	0.830313
C	3.398156	2.047947	-0.426639
C	2.961566	2.690439	-1.577485
C	2.071903	2.006073	-2.498728
C	1.121367	2.977433	-3.009496
C	-0.199344	2.609792	-3.228654
C	-3.145679	0.933786	-0.266316
C	-1.774967	5.224766	-1.163232
C	-2.823904	4.460487	-0.673249
C	-3.029235	4.346407	0.759604
C	-2.17707	5.000818	1.638037
C	-1.079342	5.799293	1.124354
C	0.459646	5.845595	-0.792836
C	0.397952	5.122597	-2.049468
C	-0.982769	4.739375	-2.278885
C	-1.274613	3.511046	-2.854805
C	-2.372553	2.710677	-2.341958
C	-3.130574	3.175897	-1.276183
C	-3.46227	2.990885	1.043366
C	-3.022454	2.348645	2.192305
C	-2.130352	3.032493	3.110937
C	-1.717717	4.329156	2.840283
C	-0.336857	4.713054	3.069633
C	0.057945	5.621241	2.008824
C	1.340945	5.560689	1.485027
C	1.546332	5.676139	0.052648
C	1.425995	4.261672	-2.406224
C	2.563086	4.084523	-1.520704

C	2.622307	4.775935	-0.31933
C	3.081261	4.104072	0.882746
C	2.289025	4.588942	1.9977
C	1.912208	3.719317	3.011109
C	0.56965	3.782812	3.559033
C	0.137822	2.427125	3.84473
C	-1.182432	2.060536	3.623826
C	-3.525576	2.267929	-0.213705
C	-2.626559	0.953987	2.137448
C	-0.883005	5.908167	-0.244478
N	0.073498	-3.729423	-2.385289

Supplementary Table 4. Atom list for optimized structure of $\mathbf{5}^{+\bullet}$ at the B3LYP/6-31G(d) level.

Symbol	X	Y	Z
N	-1.17842	0.001246	0.913907
C	-2.453691	0.002565	0.399717
C	-3.019818	1.271319	0.092133
C	-4.349158	1.227901	-0.363597
C	-5.002392	0.005224	-0.546515
C	-4.351725	-1.218821	-0.363529
C	-3.022492	-1.265012	0.092197
C	-0.765433	2.332366	0.407988
C	0.290177	3.27136	0.112013
C	-0.163548	4.519632	-0.324892
C	-1.537318	4.75346	-0.510625
C	-2.520695	3.764917	-0.351901
C	-2.155055	2.489416	0.088807
C	2.014984	1.454449	0.464959
C	3.217516	0.742591	0.134018

C	4.262259	1.553374	-0.357578
C	4.055212	2.91766	-0.56507
C	2.804445	3.540497	-0.381825
C	1.719533	2.810965	0.115408
C	2.011929	-1.458675	0.465028
C	1.713589	-2.814538	0.115415
C	2.796918	-3.546318	-0.382007
C	4.048945	-2.926108	-0.565398
C	4.258889	-1.562271	-0.357824
C	3.215922	-0.749339	0.133978
C	-0.770319	-2.330756	0.408094
C	-2.160264	-2.484916	0.088891
C	-2.528549	-3.759639	-0.351826
C	-1.547234	-4.750258	-0.510541
C	-0.172997	-4.519284	-0.324818
C	0.283315	-3.271948	0.112096
C	-0.368842	-1.107413	0.898514
C	0.964413	-0.691863	0.965618
C	0.965854	0.689847	0.965525
C	-0.366534	1.108157	0.898354
H	-4.873089	2.146393	-0.60723
H	-6.030537	0.00629	-0.893838
H	-4.87758	-2.136215	-0.607131
H	0.534664	5.317054	-0.558431
H	-3.548791	4.001989	-0.604994
H	5.225977	1.125673	-0.615212
H	2.696526	4.581592	-0.670473
H	2.686779	-4.587184	-0.670654
H	5.223451	-1.136579	-0.615623
H	-3.557127	-3.994563	-0.604973

H	0.523588	-5.318132	-0.558353
H	-1.859978	-5.733244	-0.85006
H	4.869225	-3.52583	-0.947783
H	4.87681	3.515691	-0.947274
H	-1.847987	5.737095	-0.85017

Supplementary Table 5. Atom list for optimized structure of **7** at the B3LYP/cc-pVDZ level.

Symbol	X	Y	Z
C	-0.978936	0.005783	-1.204331
C	-0.143616	4.774529	-0.107118
C	-0.125315	-4.761841	-0.117568
C	1.22747	4.512226	-0.269047
C	1.245124	-4.493211	-0.278199
C	0.683874	2.34787	-1.14364
C	0.693595	-2.329581	-1.148627
C	-1.56109	-1.260861	-0.898939
C	-1.566165	1.269335	-0.895675
C	1.110457	1.127135	-1.66034
C	1.116002	-1.105423	-1.66273
C	5.428751	2.960725	0.174264
C	5.44147	-2.917638	0.179461
C	-0.704191	2.498351	-0.84519
C	-0.693724	-2.486182	-0.85131
C	-1.08965	3.746358	-0.350466
C	-1.074666	-3.737129	-0.359538
C	3.128676	2.819376	-0.684364
C	3.140743	-2.792172	-0.688739
C	4.184816	3.551677	-0.101312
C	4.196288	-3.513907	-0.103339

C	-3.58863	-0.000718	-0.304977
C	-2.902092	-1.215648	-0.48033
C	-2.907049	1.217379	-0.47718
C	3.443938	1.475545	-1.054863
C	3.451788	-1.443657	-1.057091
C	4.6229	0.772902	-0.631853
C	4.624365	-0.739484	-0.631667
C	1.704581	3.274258	-0.748541
C	1.716826	-3.253023	-0.755282
C	2.429543	0.715262	-1.648465
C	2.432872	-0.687683	-1.650253
C	5.611685	1.57233	-0.048379
C	5.620401	-1.533993	-0.043111
H	1.948935	5.275468	0.016404
H	1.969474	-5.253914	0.006846
H	-2.137729	3.928548	-0.110651
H	-2.122305	-3.923865	-0.121284
H	4.017048	4.590715	0.176407
H	4.037756	-4.55553	0.179022
H	-3.429281	-2.143353	-0.255239
H	-3.438155	2.142391	-0.250207
H	6.541704	1.110571	0.286076
H	6.543686	-1.062733	0.288938
N	0.303526	0.008926	-1.69889
C	-5.033903	-0.004206	0.124887
C	-6.069995	-0.006052	-0.852518
C	-5.376713	-0.005429	1.504856
C	-7.406184	-0.008882	-0.423436
C	-6.736473	-0.0083	1.861547
C	-7.770013	-0.00999	0.925552

H	-8.189073	-0.010195	-1.186581
H	-6.993682	-0.009209	2.924977
C	-5.877577	-0.005223	-2.379747
H	-6.907308	-0.006357	-2.775343
C	-4.397245	-0.003093	2.692867
H	-5.057144	-0.008605	3.576716
C	-9.228454	-0.012756	1.365623
H	-9.227896	-0.013538	2.469903
C	-0.632097	-6.132833	0.381891
C	-0.655795	6.142587	0.394978
C	6.558431	-3.7897	0.797085
C	6.596561	3.768338	0.78335
C	0.511822	-7.138496	0.609281
H	1.086688	-7.322719	-0.311906
H	1.211026	-6.800133	1.390263
H	0.094346	-8.1031	0.938407
C	-1.591537	-6.733792	-0.672186
H	-1.974189	-7.710444	-0.331537
H	-2.458927	-6.081856	-0.857899
H	-1.074812	-6.885546	-1.633517
C	-1.3848	-5.957583	1.721877
H	-0.724803	-5.525153	2.490972
H	-2.260095	-5.29788	1.622001
H	-1.745138	-6.932929	2.089431
C	6.89905	-4.943059	-0.175683
H	7.690099	-5.584243	0.248235
H	6.024726	-5.580706	-0.377912
H	7.257379	-4.551256	-1.141199
C	7.851303	-2.995271	1.060182
H	8.258074	-2.553745	0.136773

H	7.697655	-2.184807	1.79017
H	8.620302	-3.666998	1.473324
C	6.080641	-4.380249	2.144412
H	5.19083	-5.017149	2.023942
H	6.873879	-5.000185	2.594905
H	5.825571	-3.580089	2.857811
C	7.818054	3.684364	-0.16199
H	8.668132	4.2499	0.25535
H	8.149669	2.645615	-0.313368
H	7.579536	4.106531	-1.151483
C	6.976028	3.185179	2.164769
H	6.122139	3.229443	2.859875
H	7.297094	2.134516	2.09622
H	7.807871	3.757801	2.608153
C	6.248448	5.255611	0.976838
H	5.979181	5.74197	0.026067
H	5.41503	5.396237	1.683195
H	7.120281	5.78932	1.387083
C	0.484048	7.152521	0.623739
H	1.185165	6.815362	1.403485
H	1.057662	7.340934	-0.297357
H	0.062752	8.114711	0.955071
C	-1.407246	5.961927	1.734949
H	-1.771617	6.935117	2.104262
H	-2.279737	5.298629	1.634395
H	-0.745041	5.53111	2.503055
C	-1.618137	6.741402	-0.657674
H	-2.482785	6.08611	-0.844334
H	-2.00483	7.715809	-0.315141
H	-1.102344	6.897091	-1.618879

C	-3.529438	-1.268933	2.822398
H	-4.13718	-2.182434	2.718905
H	-2.725301	-1.303431	2.074054
H	-3.05428	-1.292718	3.817626
C	-3.54411	1.272228	2.827132
H	-2.741172	1.319105	2.078106
H	-4.162522	2.179018	2.72797
H	-3.068144	1.29743	3.821944
C	-5.218472	-1.276145	-2.947427
H	-4.141232	-1.319607	-2.7342
H	-5.686882	-2.185852	-2.538193
H	-5.33978	-1.29957	-4.043627
C	-5.221527	1.267536	-2.946789
H	-5.69209	2.175918	-2.537086
H	-4.144397	1.313547	-2.733619
H	-5.342994	1.291205	-4.042965
C	-9.965234	-1.282661	0.90384
H	-9.458073	-2.192198	1.262882
H	-10.999793	-1.293564	1.285396
H	-10.015787	-1.339561	-0.196427
C	-9.969301	1.255445	0.905633
H	-9.465105	2.166079	1.266061
H	-10.01989	1.313792	-0.194559
H	-11.003932	1.262453	1.287092

Supplementary Table 6. Atom list for transition state of bowl inversion of **7** at the B3LYP/cc-pVDZ level.

Symbol	X	Y	Z
C	-1.042475	0.006247	0.025597
C	-0.169146	4.964181	0.058964

C	-0.142775	-4.946572	-0.025699
C	1.220434	4.693108	0.045861
C	1.245693	-4.667409	-0.034866
C	0.660378	2.401746	0.033631
C	0.673559	-2.379228	-0.011861
C	-1.637915	-1.299786	0.018857
C	-1.645303	1.309009	0.042561
C	1.088127	1.107873	0.018918
C	1.095091	-1.082572	-0.003523
C	5.650967	3.09922	-0.006219
C	5.672304	-3.04329	-0.056599
C	-0.762277	2.572628	0.046349
C	-0.747744	-2.557971	-0.001366
C	-1.141894	3.918693	0.059076
C	-1.120323	-3.906482	-0.009357
C	3.192102	2.914761	0.015705
C	3.208531	-2.878221	-0.03626
C	4.368688	3.697549	0.010854
C	4.387065	-3.648209	-0.05194
C	-3.733157	-0.001647	0.052968
C	-3.043429	-1.237495	0.03068
C	-3.05012	1.238223	0.059073
C	3.482707	1.511047	0.001818
C	3.493016	-1.470042	-0.026144
C	4.737621	0.80069	-0.016069
C	4.741302	-0.756412	-0.029723
C	1.725392	3.374848	0.032112
C	1.742499	-3.346817	-0.028532
C	2.382128	0.701552	0.004378
C	2.386316	-0.668208	-0.009584

C	5.827247	1.680302	-0.019387
C	5.842325	-1.629368	-0.045784
H	1.917517	5.529942	0.046061
H	1.947289	-5.500424	-0.046803
H	-2.19919	4.189399	0.069243
H	-2.176274	-4.18261	-0.002962
H	4.293973	4.784236	0.020003
H	4.325681	-4.738344	-0.060605
H	-3.635839	-2.153942	0.012744
H	-3.647679	2.150957	0.090646
H	6.845494	1.286639	-0.032813
H	6.85331	-1.224242	-0.049959
N	0.301082	0.010133	0.013815
C	-5.241957	-0.004976	0.08024
C	-5.984876	0.096634	-1.130509
C	-5.934773	-0.108434	1.317171
C	-7.386846	0.091429	-1.066448
C	-7.340301	-0.106002	1.306163
C	-8.091887	-0.008553	0.135781
H	-7.942317	0.167189	-2.005043
H	-7.867013	-0.18423	2.262097
C	-5.400694	0.222784	-2.549465
H	-6.28532	0.154909	-3.205119
C	-5.300246	-0.254008	2.712425
H	-6.150147	-0.123883	3.403671
C	-9.614729	-0.015907	0.177219
H	-9.903662	-0.089199	1.240506
C	-0.658536	-6.406468	-0.033492
C	-0.693297	6.421034	0.07414
C	6.903797	-3.98432	-0.073673

C	6.931893	3.970203	-0.011389
C	0.480999	-7.442069	-0.04691
H	1.114843	-7.347626	-0.942614
H	1.125446	-7.359063	0.842369
H	0.054576	-8.457519	-0.050955
C	-1.524633	-6.643045	-1.293492
H	-1.901489	-7.679443	-1.31133
H	-2.39705	-5.972601	-1.329386
H	-0.937605	-6.477349	-2.211208
C	-1.511476	-6.661854	1.231882
H	-0.91504	-6.509295	2.145813
H	-2.383829	-5.992734	1.286566
H	-1.887655	-7.698623	1.238458
C	6.864024	-4.868168	-1.342824
H	7.735693	-5.543554	-1.36856
H	5.958659	-5.492845	-1.383298
H	6.884194	-4.249136	-2.254198
C	8.241084	-3.219746	-0.077951
H	8.347476	-2.576671	-0.965758
H	8.361862	-2.591972	0.818917
H	9.075309	-3.938895	-0.090738
C	6.883601	-4.887954	1.181965
H	5.978601	-5.512855	1.227077
H	7.755158	-5.564	1.183475
H	6.918582	-4.283135	2.102397
C	7.760244	3.667299	-1.282431
H	8.675546	4.282537	-1.300938
H	8.068961	2.612035	-1.333742
H	7.180061	3.890434	-2.192266
C	7.7801	3.652203	1.24278

H	7.21458	3.865394	2.164167
H	8.088423	2.596107	1.277083
H	8.696194	4.266481	1.253853
C	6.628314	5.480252	-0.000105
H	6.053306	5.793311	-0.885815
H	6.068952	5.783181	0.899027
H	7.573961	6.045016	-0.005111
C	0.439997	7.463536	0.071875
H	1.087752	7.372038	0.957879
H	1.071721	7.385551	-0.826913
H	0.007439	8.476354	0.083376
C	-1.543174	6.652558	1.34611
H	-1.925373	7.686887	1.36946
H	-2.411372	5.977435	1.393926
H	-0.942554	6.489863	2.255533
C	-1.565323	6.671328	-1.179155
H	-2.43439	5.996976	-1.221869
H	-1.947716	7.705851	-1.18038
H	-0.980791	6.522361	-2.10133
C	-4.755939	-1.669626	2.985851
H	-5.501879	-2.440143	2.732752
H	-3.840697	-1.875024	2.411357
H	-4.506301	-1.778147	4.054919
C	-4.27606	0.821062	3.117004
H	-4.111821	0.77682	4.206866
H	-3.300437	0.68153	2.632155
H	-4.637449	1.833256	2.874399
C	-4.472556	-0.921719	-2.994518
H	-3.48044	-0.866622	-2.526004
H	-4.908502	-1.906218	-2.759832

H	-4.325237	-0.87242	-4.086603
C	-4.772257	1.597619	-2.84833
H	-5.440108	2.417962	-2.539558
H	-3.805795	1.730715	-2.341061
H	-4.592003	1.699287	-3.93193
C	-10.200604	-1.241932	-0.545826
H	-9.803655	-2.180411	-0.127362
H	-11.299175	-1.262322	-0.451804
H	-9.958633	-1.226412	-1.621799
C	-10.213053	1.290376	-0.374237
H	-9.822606	2.16812	0.164787
H	-9.976385	1.421141	-1.443543
H	-11.311312	1.288146	-0.275106

Supplementary Table 7. Summary of calculations on the bowl-to-bowl inversion process of **7**.

	**ground state**	**transition state**
Energy (au)	–2572.302341	–2572.271074
Total Energy (au)	–2572.398423	–2572.366659
E_a (kcal/mol)	0	19.9
